# Personality changes related to presence and treatment of substance use (disorders): a systematic review

**DOI:** 10.1017/S003329172400093X

**Published:** 2024-07

**Authors:** Christina M. Juchem, Antonia Bendau, Leonie C. Bandurski, Nico J. Reich, Saskia Baumgardt, Eva Asselmann

**Affiliations:** 1 Department of Psychology, Institute for Mental Health and Behavioral Medicine, HMU Health and Medical University, Potsdam, Germany; 2 Department of Psychiatry and Neurosciences, Charité – Universitätsmedizin Berlin, Corporate Member of Freie Universität Berlin and Humboldt Universität zu Berlin, CCM, Charitéplatz 1, 10117 Berlin, Germany

**Keywords:** addiction, drugs, misuse, personality development, personality traits

## Abstract

Heavy substance use (SU) and substance use disorders (SUD) have complex etiologies and often severe consequences. Certain personality traits have been associated with an increased risk for SU(D), but far less is known about personality changes related to SU(D). This review aims to synthesize the existing literature on this research question. A systematic literature search was conducted from November 2022 to February 2023 in PubMed, EbscoHost, and Web of Science. Peer-reviewed original papers on SU(D)-related personality changes were included. Of 55 included studies, 38 were observational population-based studies and 17 were intervention studies. Overall, personality and SU measures, samples, study designs, and statistical approaches were highly heterogenous. In observational studies, higher SU was most consistently related to increases in impulsivity-related traits and (less so) neuroticism, while interventions in the context of SU(D) were mostly associated with increases in conscientiousness and self-efficacy and lasting decreases in neuroticism. Findings for traits related to extraversion, openness, conscientiousness, and agreeableness were mixed and depended on SU measure and age. Studies on bidirectional associations suggest that personality and SU(D) both influence each other over time. Due to their strong association with SU(D), impulsivity-related traits may be important target points for interventions. Future work may investigate the mechanisms underlying personality changes related to SU(D), distinguishing substance-specific effects from general SU(D)-related processes like withdrawal, craving, and loss of control. Furthermore, more research is needed to examine whether SU(D)-related personality changes vary by developmental stage and clinical features (e.g. initial use, onset, remission, and relapse).

## Introduction

Heavy substance use (SU) and substance use disorders (SUD) are highly prevalent conditions that can have severe consequences for individuals, their families, and society (Gowing et al., 2015). According to vulnerability/diathesis-stress-models (Whelan et al., 2014; Wittchen et al., 2014; Zuckerman, 2000), SU(D) results from interactions between environmental stressors, psychosocial characteristics, and individual vulnerabilities, including specific personality traits (Kotov, Gamez, Schmidt, & Watson, 2010; Nevid, Gordon, Miele, & Keating, 2020).

Personality traits are relatively stable and enduring patterns of thoughts, feelings, and behaviors that differ between individuals (Kandler, Zimmermann, & McAdams, 2014). A large proportion of these differences can be described by the Big Five traits extraversion, agreeableness, conscientiousness, neuroticism, and openness to experience (McCrae & Costa, 2008). Extraversion refers to one's sociability, assertiveness, and preference for social interactions. Neuroticism reflects negative affect, emotional instability, and insecurity. Openness includes intellect and willingness to explore new experiences. Conscientiousness refers to orderliness, responsibility, and self-discipline. Agreeableness refers to cooperativeness, empathy for others, and kindness. The Big Five are linked to life outcomes in various domains including health (Strickhouser, Zell, & Krizan, 2017). For example, individuals high in neuroticism are more susceptible to stress and detrimental coping strategies like substance use (Connor-Smith & Flachsbart, 2007), and individuals low in agreeableness may be less likely to comply with legal regulations, increasing the likelihood of illicit drug use (Dash, Martin, & Slutske, 2023). More broadly, personality also subsumes other traits, such as self-esteem or impulsivity. The terms *impulsivity* and *impulsiveness* are often used interchangeably in the literature yet their precise definition may vary by context. Here, we use the more common term *impulsivity,* which describes the tendency to act on impulse and without considering the consequences. Impulsivity overlaps with other personality measures. For example, the frequently used UPPS Impulsive Behavior scale (Whiteside & Lynam, 2009) includes four facets that relate to specific facets of the Big Five: Urgency (neuroticism), perseverance and premeditation (conscientiousness), and sensation seeking (extraversion) (Kandler et al., 2014).

Although personality traits are relatively stable in the short term, they can – and do – change over time. Previous studies have shown that personality develops across the entire lifespan and in relation to major life experiences (Bleidorn, Hopwood, & Lucas, 2018; Denissen, Luhmann, Chung, & Bleidorn, 2019). Theoretical framework (e.g. TESSERA) assume that personality may change due to repeated sequences of triggering situations, expectancies, state expressions, reactions, and associative/reflective processes (Wrzus & Roberts, 2017). These components may be affected by SU(D). For example, individuals with SUD often invest increasing time in obtaining/using drugs and neglect other areas of life (e.g. social relationships and work), which might lower conscientiousness. Moreover, substance-specific physiological effects may lead to changes in personality traits. Taken together, personality might affect whether individuals engage in SU (selection effects). At the same time, personality might change due to SU (socialization effects).

Consistently, different models on the relationship between SU and personality have been proposed (Klimstra, Luyckx, Hale, & Goossens, 2014; Samek et al., 2018). The *vulnerability model* suggests that certain personality traits (e.g. high neuroticism) predispose to SUD. Conversely, the *scar model* posits that SUD leads to personality changes (e.g. increasing neuroticism). The *common cause model* posits that SUD and specific personality trait levels (e.g. high neuroticism) stem from shared etiological factors but do not directly influence each other. The *transactional model* suggests that SUD and personality bidirectionally affect each other over time (Samek et al., 2018). The *spectrum model* suggests that personality traits and (problematic) behaviors lie on the same continuum (Klimstra et al., 2014; Krueger et al., 2021; Krueger & Tackett, 2003). For example, SU could be a manifestation of neuroticism, so that increasing neuroticism should be correlated with increasing SU.

The predictive role of personality traits in SUD has been widely studied (for a meta-analysis see Kotov et al., 2010). In line with the vulnerability model, numerous studies have shown that certain trait levels, especially high neuroticism, impulsivity, and sensation seeking, as well as low self-directedness and harm avoidance, relate to an increased risk for SUD (Dash et al., 2019; Kotov et al., 2010; Nevid et al., 2020; Sher, Bartholow, & Wood, 2000; Whiteside & Lynam, 2009). In contrast, much less is known about personality changes associated with SU(D) (Blonigen et al., 2015; Kroencke, Kuper, Bleidorn, & Denissen, 2021). Different substances might also be associated with different (changes in) personality traits due to substance-specific physiological mechanisms, social norms, and legal regulations (e.g. alcohol *v.* illicit drugs) (Robinson & Adinoff, 2016).

Research on SU(D)-related personality changes is important for several reasons. First, it may identify important mechanisms underlying personality development. For example, how does SU affect personality, and how might these effects explain age-graded personality changes (e.g. during adolescence)? Second, it may improve knowledge concerning the impact of personality changes on SU(D). To illustrate, does increasing neuroticism lead to heavier drinking? Examining such questions may provide predictive markers of symptom progression and recovery, which are needed for early detection and targeted intervention (Choi et al., 2023; Conrod, 2019; Debenham et al., 2021; Kroencke et al., 2021). Third, findings on personality changes due to SU(D) interventions may elucidate indicators of treatment success (Blonigen & Macia, 2021; Hershberger, Um, & Cyders, 2017).

Despite its importance, research on SU(D)-related personality changes is fragmented and lacks a comprehensive synthesis (Kroencke et al., 2021; Nevid et al., 2020). Thus, this systematic review aims to summarize existing evidence in this field by addressing the following research questions: (1) How does personality change before, during, and after the onset or remission of SU(D) (within-person comparisons)? (2) How are personality changes related to changes in diagnostic features (e.g. symptom severity) of SU(D) (within-person comparisons)? (3) Do personality changes differ between individuals with *v.* without SU(D) (between-person comparisons)?

## Methods

### Literature search

This review was preregistered in the PROSPERO Systematic Reviews Database (CRD42022370973) and follows the PRISMA and other best practice guidelines for systematic reviews (Page et al., 2021; Siddaway, Wood, & Hedges, 2019).

A systematic literature search was conducted on EBSCOhost, PubMed, and Web of Science (11/2022-01/2023). Additionally, literature cited in the identified papers was manually retrieved for further analysis. The review was restricted to peer-reviewed original papers published in academic journals in either English or German language, focusing exclusively on studies in humans. No restrictions were applied regarding the date of publication or other formal aspects of the search. Search terms were applied to the title, keywords, and abstract of potential studies. To ensure inclusion of the most recent studies, the search was repeated prior to the final submission.

**Figure 1.**
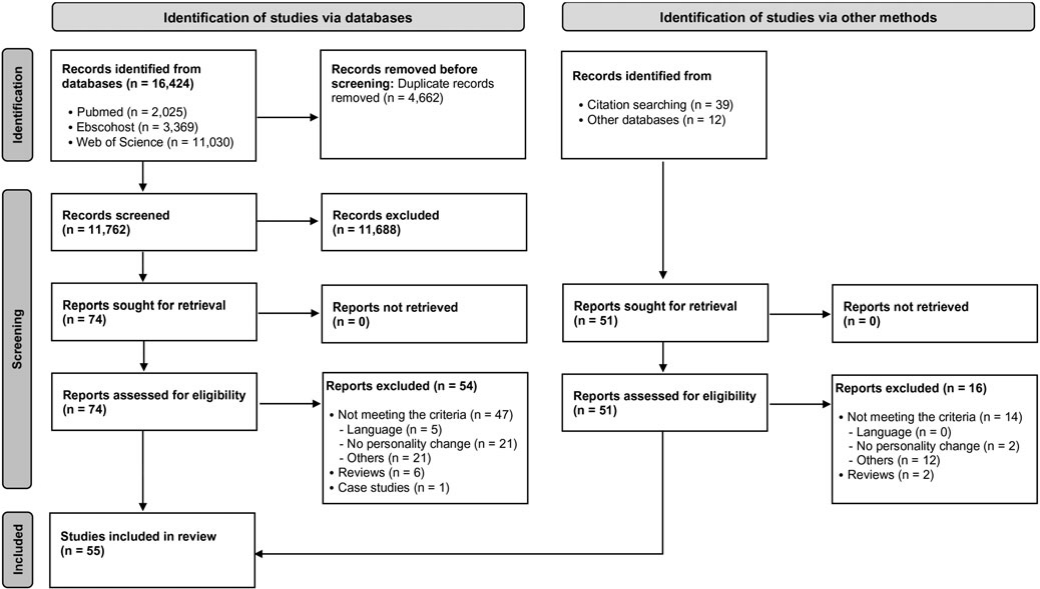
PRISMA flow diagram.

Search terms (online Supplementary Table S1) included a combination of personality (e.g. Big Five domains, self-efficacy, impulsivity), change (e.g. change, development), and SU/addiction (e.g. addiction, substance use disorder, heavy drinking) keywords. The PRISMA flowchart (Fig. 1) depicts the procedure of the search, screening, and data extraction.

### Screening and data extraction

To select the studies for inclusion, 2 reviewers applied the eligibility criteria. One reviewer screened the records and selected the studies based on the inclusion/exclusion criteria, while the other reviewer double-checked these decisions. Disagreements were resolved by consulting a third independent reviewer and finding a consensus decision. The same procedure was carried out for extracting and checking the retrieved data. The reviewers followed a standardized protocol with several steps, including examination of the titles and abstracts of references obtained from the databases and reading the full texts of potentially relevant articles. Of the studies meeting inclusion criteria, the aims, methods (e.g. country, sample, design, outcome variables), results, limitations, and potential biases were recorded and summarized. Given the heterogeneity of personality traits, SU measures, samples, study designs, and statistical approaches, a narrative synthesis rather than a meta-analysis was conducted.

## Results

Of the 55 studies that met the inclusion criteria (publication dates: 1986–2022), 38 were observational studies on SU-related personality changes in population samples (Table 1) and 17 were (clinical) intervention studies in individuals with or at high risk for SU(D) (Table 2). Because the literature search yielded a plethora of personality constructs, we created eight clusters based on conceptual overlap among them: extraversion-related traits, neuroticism-related traits, openness-related traits, conscientiousness-related traits, agreeableness-related traits, impulsivity-related traits, self-efficacy/self-esteem-related traits, and other. See online Supplementary Table S3 for more information. We did this to better structure the results and to identify similarities and differences in the findings.

**Table 1.** Observational population-based studies

Author year	Country	Sample	Study design	SU	SU measure	Personality construct	Key findings
Allen et al. (2015)	Australia	*N* = 13 301 adults from a nationally representative sample (*M* = 44 at wave 1)^a^	Longitudinal, 2 waves across 4 years	Alcohol, nicotine	Frequency and quantity of SU^b^	Extraversion, neuroticism, openness, conscientiousness, agreeableness	Over the study period, higher alcohol use at baseline was linked to increases in neuroticism. Increase in alcohol use from baseline to follow-up was linked to a simultaneous increase in neuroticism. Higher alcohol use at baseline was linked to lower intraindividual stability of extraversion. Over the study period, higher nicotine use at baseline was linked to decreases in agreeableness and conscientiousness. Increases in nicotine use from baseline to follow-up were linked to simultaneous decreases in extraversion. Higher nicotine use at baseline was linked to lower intraindividual stability of neuroticism over the study period. There were no significant associations between SU and changes in any other personality traits.
Ashenhurst et al. (2015)	United States	*N* = 2245 university students (*M* = 18 years at wave 1)	Longitudinal, 10 waves across 6 years, 3 waves of personality assessment (end of high school, end of college, 2 years after college)	Alcohol	Frequency of binge drinking	Impulsivity, sensation seeking	Seven binge drinking trajectories were identified: frequent, moderate, increasing, occasional, low increasing, decreasing, and rare. From high school through the end of college, the occasional, decreasing, and rare group decreased in sensation seeking, whereas the increasing group increased in sensation seeking. The moderate group decreased in impulsivity, whereas the frequent group increased in impulsivity. From the end of college through two years later, the frequent group decreased in impulsivity, whereas the increasing group increased in impulsivity. There were no significant associations for any other group and changes in sensation seeking or impulsivity.
Blonigen et al. (2015)	United States	*N* = 4968 twins from adolescence to young adulthood (10–32 years)^c^	Longitudinal, 4 waves across 15 years	Alcohol	Initiation of alcohol use	Positive emotionality (i.e. well-being), negative emotionality (i.e. stress reaction, alienation, and aggression), constraint (i.e. control and harm avoidance)	Alcohol use initiation was linked to decreases in positive emotionality and constraint, and it was linked to increases in negative emotionality.
Chassin et al. (2010)	United States	*N* *=* 1170 male adolescent juvenile offenders (14–17 years at wave 1)	Longitudinal, 6 waves across 3 years	Alcohol, cannabis	Frequency of SU^b^	Psychosocial maturity (i.e. responsibility, temperance, and perspective taking)	^d^Over the study period, there was no significant association between change in alcohol use and change in psychosocial maturity, but increasing cannabis use was linked to a lower increase in psychosocial maturity over the study period. Examining lagged effects across one wave, both higher alcohol and cannabis use predicated decreased psychosocial maturity six months later, but only for cannabis the reverse was also significant. Moreover, four trajectory groups were identified for alcohol use (i.e. adolescence-limited, low, decreasing, and increasing) and cannabis use (i.e. decreasing, low, increasing, and late adolescent onset), respectively. Over the entire study period, decreasing alcohol use was linked to increasing psychosocial maturity. Increasing alcohol use was linked to decreases in psychosocial maturity. Decreasing cannabis use was linked to increasing psychosocial maturity. Late adolescent onset of cannabis use was linked to decreases in psychosocial maturity. There were no significant associations between any other SU trajectories and change in psychosocial maturity.
De Moor et al. (2022)	Netherlands	*N* *=* 360 adolescents (11–18 years at wave 1)	Longitudinal, 3 waves across 3 years	Alcohol, nicotine, cannabis	Frequency of SU^b^	Identity status (i.e. educational and relational identity)	^d^Three clusters of identity status were identified at each time point: Achievement, moratorium, and diffusion (characterized by different levels of commitment and exploration based on the identity status approach). Over the entire period, three types of transitions between identity clusters were found: stable, progressive, and regressive (with maturation understood as moving towards an identity status characterized by low exploration and high commitment). Overall, there were no significant associations between transition types over the entire study period and SU at wave 3. Post hoc tests revealed that individuals with a stable *v.* a regressive transition in educational identity were more likely to abstain from alcohol use at wave 2 (wave 3 data was not available).
De Win et al. (2006)	Netherlands	*N* *=* 188 young adults (18–35 years at wave 1)	Longitudinal, 2 waves across 17 months	Ecstasy	Frequency and quantity of SU	Impulsivity, sensation seeking	Higher ecstasy use at wave 1 was linked to higher sensation seeking at T2, adjusted for sensation seeking at wave 1. There was no significant association between ecstasy use at wave 1 and impulsivity at wave 2, adjusted for ecstasy use at wave 1.
Gmel et al. (2020)	Switzerland	*N* = 5125 young male adults (20 years at wave 1)	Longitudinal 2 waves across 6 years	Alcohol	Quantity of SU, frequency of binge drinking	Aggression-hostility, sociability, neuroticism-anxiety, sensation seeking	^d^Over the study period, higher alcohol use at wave 1 was linked to increases in aggression, sociability, and sensation seeking from wave 1 to 2, whereas only aggression and sensation seeking at wave 1 were linked to increases in alcohol use from wave 1 to 2. Higher binge drinking at baseline was linked to increases in sensation seeking from wave 1 to 2, whereas aggression, sociability, and sensation seeking at baseline were linked to increases in binge drinking from wave 1 to 2. There were no significant associations between alcohol use and change in neuroticism-anxiety in either direction.
Hakulinen and Jokela (2019)	Germany, United States, Australia	*N* = 39 722 adults (from 6 pooled cohort studies with nationally representative samples, *M* *=* 51 years, s.d. = 12.85 at wave 1)^a^,^e^,^f^,^g^,^h^	Longitudinal, 2 waves across *M* *=* 5.6 years	Alcohol	Risky alcohol use (i.e. heavy alcohol consumption, frequency of binge drinking, alcohol-related problems)	Extraversion, neuroticism, openness, conscientiousness, agreeableness	Risky alcohol use at wave 1 was linked to higher extraversion and neuroticism, and lower agreeableness, and conscientiousness at wave 2, adjusted for the same variables at wave 1. Individuals who changed from risky alcohol use to non-risky alcohol use from wave 1 to 2 simultaneously decreased in extraversion and increased in agreeableness and conscientiousness. Except for extraversion, these findings were consistent across cohort studies. Alcohol use was not significantly linked to changes in openness.
Hemphälä, Kosson, Westerman, and Hodgins (2015)	Sweden	*N* *=* 180 adolescents and their parents (*M* = 17 years, s.d. = 1.9)	Longitudinal, 2 waves across 5 years	*Any*	SUD	Psychopathic traits (i.e. 4 facets: interpersonal, affective, lifestyle, antisocial)	^i^Rank-order correlations were moderate to high for all psychopathic traits and facets. For both women and men, there were decreases over the study period in total psychopathic trait scores, as well as in the interpersonal, affective, and lifestyle facets. There was no significant change in the antisocial facet.
Hicks et al. (2012)	United States	*N* *=* 2182, male and female adolescent twins (11 years at wave 1)^c^	Longitudinal, 3 waves across 13 years (at ages 11, 17, and 24)	Alcohol	AUD	Constraint (reverse behavioral disinhibition), negative emotionality, positive emotionality	Four groups describing onset and course of AUD were identified: Never onset, adult onset, adolescent onset-desist, adolescent onset-persist. (Due to differences in measures, personality at age 11 could not be compared to later waves.) Adult onset was linked to a lower decrease in negative emotionality compared to the Never onset group. Adolescence onset-persist was linked to the lowest decreases compared to all other groups. Adult onset and adolescent-onset persist was linked to a lower decrease in behavioral disinhibition compared to the never onset group. Adolescent onset-desist was linked to a greater decrease in behavioral disinhibition compared to the adult onset and the adolescent onset-persist group. There was no change in positive emotionality over the study period and no significant association with AUD to begin with, so this trait was excluded from subsequent analyses.
Horvath, Milich, Lynam, Leukefeld, and Clayton (2004)	United States	*N* *=* 481 adolescents (15 years at wave 1)	Longitudinal, 2 waves across 5 years (at ages 15 and 20)	Alcohol, nicotine, cannabis	Frequency of SU^b^	Sensation seeking	^d^Higher SU at wave 1 was linked to increased sensation seeking at wave 2, while in return, higher sensation seeking at wave 1 was linked to increased SU at wave 2. Findings were similar for alcohol, tobacco, and cannabis use.
Jokela et al. (2018)	Australia, Japan, United Kingdom, United States, Germany	*N* = 56 786 adults (from 7 cohort studies, *M* *=* 51 years, s.d. = 12.61)^a^,^e^,^f^,^g^,^h^,^j^	Longitudinal, 3 waves across 4–18 years	Alcohol, nicotine	Smoking status, quantity of SU, heavy alcohol consumption, binge drinking^b^	Extraversion, neuroticism, openness, conscientiousness, agreeableness	Within-person increases in extraversion were linked to within-person increases in alcohol consumption when all personality traits were fitted in one model. When fitted separately for each trait, increases in extraversion were also related to increases in smoking, and increases in conscientiousness were linked to decreases in alcohol consumption. There were no significant findings for changes in openness, agreeableness and neuroticism in relation to changes in SU.
Kaiser et al. (2016)	United States	*N* *=* 525 college students (*M* *=* 19 years at wave 1)^k^	Longitudinal, 2 waves across 2 years	Alcohol	Frequency and quantity of alcohol use	Impulsive personality traits (i.e. negative urgency, positive urgency, sensation seeking, lack of premeditation, lack of perseverance)	^d^Higher positive urgency and lack of perseverance at wave 1 were linked to increased alcohol use at wave 2. In return, higher alcohol use at baseline was linked to increased negative urgency, positive urgency, lack of premeditation, and sensation seeking at wave 2.
Kaiser et al. (2018)	United States	*N* *=* 525 college students (*M* *=* 19 years at wave 1)^k^	Longitudinal, 3 waves across 3 years	Alcohol	Frequency and quantity of alcohol use, alcohol problems	Impulsive personality traits (i.e. negative urgency, sensation seeking, lack of premeditation)	^d^Over the study period, higher sensation seeking was linked to increased alcohol use at the following waves. In return, higher alcohol use was linked to increased negative urgency, sensation seeking, and lack of premeditation at the following waves. Thus, only sensation seeking and alcohol use showed bidirectional associations. Moreover, sensation seeking at wave 2 was a mediator between alcohol use at wave 1 and 3. Alcohol use/problems at wave 2 was a mediator between sensation seeking at wave 1 and 3, and between alcohol use at wave 1 and negative urgency at wave 3.
Klimstra et al. (2014)	Belgium	*N* *=* 485 university students (*M* *=* 19 years at wave 1)	Longitudinal, 4 waves across 4 years	Alcohol, cannabis	Alcohol abuse (i.e. drunkenness), cannabis use frequency	Extraversion, neuroticism, openness, conscientiousness, agreeableness (coupled with 3 facets, respectively)	^d^Over the study period, there was positive correlated rank-order change between alcohol abuse and extraversion as well as two of its facets, and negative correlated change with conscientiousness and one of its facets. There was positive correlated rank-order change between cannabis use and one facet of extraversion, and negative correlated change with conscientiousness. There were no significant associations between rank-order changes in SU and any other personality traits or facets. Higher alcohol abuse was linked to higher extraversion and one of its facets and one facet of openness at following waves, and it was linked to lower agreeableness and conscientiousness and one of its facets. Cannabis use was linked to lower extraversion and one of its facets, and it was linked to higher openness and two of its facets at following waves. There were no significant associations for any other personality traits or facets predicting later SU. Openness and one of its facets were linked to higher cannabis use at the following waves. Conscientiousness was linked to lower cannabis use at the following waves. There were no significant associations between any other personality trait or facet predicting later SU.
Kroencke et al. (2021)	Netherlands	*N* *=* *10 872* adults from a nationally representative sample (*M* *=* 50 years, s.d. *=* 17.2)	Longitudinal, *M* = 4.25 waves across 10 years	Alcohol, nicotine, sedatives, soft drugs, ecstasy, hallucinogens, hard drugs	Smoking status, heavy alcohol use, frequency of SU for sedatives, soft drugs, ecstasy, hallucinogens, and hard drugs^b^	Extraversion, neuroticism, openness, conscientiousness, agreeableness, self-esteem	Within-person increase in nicotine use was linked to a within-person decrease in neuroticism at the following wave. Increased sedative use was linked to increases in neuroticism and decreases in self-esteem at the following wave. Increased use of hard drugs was linked to decreases in agreeableness. There were no significant associations with extraversion, conscientiousness, and openness and no significant associations between other substances and changes in any personality traits.
Labouvie and McGee (1986)	United States	*N* = 882 adolescents (12–18 at wave 1)	Longitudinal, 2 waves across 3 years	Alcohol, nicotine, cannabis, cocaine	Frequency and quantity of SU^l^	Achievement, affiliation, autonomy, cognitive structure, exhibition, harm-avoidance, impulsivity, play, self-esteem	Over the study period, three SU groups were identified: light, moderate, heavy. Affiliation, autonomy, exhibition, impulsivity, and play were linked to higher SU, whereas achievement, cognitive structure, and harm-avoidance were linked to lower SU. Across user groups, there were no significant differences in mean changes of personality traits over the study period. There were no significant associations for self-esteem and nine other personality traits which were not reported.
Littlefield et al. (2009)	United States	*N* *=* 489 college students (*M* *=* 18 years at wave 1)^m^	Longitudinal, 7 waves across 17 years (at ages 18, 19, 20, 21, 25, 29, and 35)	Alcohol	Problematic alcohol use (i.e. negative consequences of drinking, symptoms related to alcohol dependence)	Impulsivity, extraversion, neuroticism	Over the study period, decreases in problematic alcohol use were linked to decreases in impulsivity and neuroticism. There were no significant associations between change in problematic alcohol use and change in extraversion.
Littlefield et al. (2010b)	United States	*N* *=* 489 college students (*M* *=* 21 years at wave 1)^m^	Longitudinal, 4 waves, across 14 years (at ages 21, 25, 29, and 35)	Alcohol	Problematic alcohol use (i.e. negative consequences of drinking, symptoms related to alcohol dependence)	Extraversion, neuroticism, openness, conscientiousness, agreeableness	Over the study period, increases in problematic alcohol use were linked to increases in neuroticism and decreases in conscientiousness. There were no significant associations between change in problematic alcohol use and change in extraversion, agreeableness, and openness.
Littlefield et al. (2010a)	United States	*N* *=* 489 college students (*M* *=* 18 years at wave 1)^m^	Longitudinal, 7 waves across 17 years (at ages 18, 19, 20, 21, 25, 29, and 35)	Alcohol	Frequency of alcohol use, heavy drinking, alcohol-related problems	Impulsivity	Over the study period, five trajectories of impulsivity were identified. From age 18 to 25, the group with the trajectory of steepest decrease in impulsivity was linked to steeper decreases in alcohol use and heavy drinking compared to the other groups. From age 25 to 35, the two groups characterized by modest decreases in impulsivity were linked to steeper decreases in alcohol use compared to the group with highest decreases in impulsivity, but the latter group showed steeper decreases for the measure of heavy drinking. There was no significant interaction between trajectory and alcohol related problems.
Littlefield and Sher (2012)	United States	*N* *=* 489 college students (*M* *=* 18 years at wave 1)^m^	Longitudinal, 4 waves across 17 years (at ages 18, 25, 29, and 35)	Nicotine	Smoking status, Frequency and quantity of nicotine use, Perceived tobacco dependence, Tobacco use disorder	Neuroticism, impulsivity	Four groups of nicotine use patterns were identified: abstainers, onsetters, desisters, and persisters. From ages 18 to 25, omnibus time by group interactions were found for neuroticism but not for impulsivity. Contrast analysis was only conducted for desisters and revealed that this group exhibited greater decreases in neuroticism and impulsivity compared to all other groups. From ages 18 to 35, omnibus time by group interactions were found for impulsivity but not for neuroticism. For neuroticism, contrast analysis was only conducted for desisters and showed no significant association. For impulsivity, contrast analyses were conducted for all groups and revealed that persisters exhibited greater decreases in impulsivity compared to all other groups.
Littlefield et al. (2012)	United States	*Sample 1: N* *=* 3720 college students, *Sample 2: N* *=* 489 college students (*M* *=* 18 years at wave 1)^m^	Longitudinal, *Sample 1:* (a) 3 waves (fall freshmen, spring sophomore, spring senior year), (b) 3 waves (fall freshmen, fall senior, post-college) *Sample 2:* 4 waves across 17 years (at ages 18, 25, 29, 35)	Alcohol	Frequency of alcohol use, alcohol intoxication, and heavy drinking; problematic alcohol use (i.e. negative consequences of drinking, symptoms related to alcohol dependence)	Impulsivity, novelty seeking	^d^*Sample 1:* From fall freshmen to spring senior year, increases in novelty seeking were related to increases in alcohol use, but no transactional pathways were found i.e., neither from novelty seeking at wave 1 to change in alcohol use nor from alcohol use at wave 1 to change in novelty seeking. From fall freshmen to spring sophomore year, higher levels of novelty seeking were linked to increases in alcohol use, and from spring sophomore to spring senior year, higher levels of heavy drinking were linked to increases in novelty seeking. From fall freshmen to fall senior year but not from fall senior year to post-college, increases in novelty seeking were linked to increases in alcohol use. There were no transactional pathways from novelty seeking at wave 1 to change in alcohol use nor from alcohol use at wave 1 to change in novelty seeking, except for novelty seeking at fall senior year being linked to increases in alcohol use from fall senior year to post-college. *Sample 2:* Over the study period, increases in problematic alcohol use were linked to increases in impulsivity, but no transactional pathways were found, that is, neither from impulsivity at wave 1 to change in problematic alcohol use nor from problematic alcohol use at wave 1 to change in impulsivity.
Luchetti et al. (2018)	United States	*N* *=* *10 094* adults from a nationally representative sample (*M* *=* 69 years, s.d. *=* 9.97)^e^	Longitudinal, 2 waves across 4 years	Alcohol	Frequency of alcohol use, alcohol use disorder	Extraversion, neuroticism, openness, conscientiousness, agreeableness	Participants were clustered into 3 alcohol use groups: Non-drinkers, Light-to-moderate drinkers, and Moderate-to-heavy drinkers. Over the study period, Light-to-moderate drinkers exhibited a lower decrease in extraversion compared to Non-drinkers. In addition, individuals with a history of alcohol dependence exhibited a greater decrease in conscientiousness. There were no significant associations between any other alcohol use groups and change in any other personality traits.
Malmberg et al. (2013)	Netherlands	*N* *=* 1068 adolescents (12–13 years at wave 1)	Longitudinal, 4 waves across 32 months (baseline, 8 months, 20 months, 32 months)	Alcohol, nicotine	Frequency of alcohol use, Binge drinking, Frequency of nicotine use^b^	Impulsivity, sensation seeking, anxiety sensitivity, hopelessness	^d^Over the study period, there was one significant association between SU and anxiety sensitivity, that is, higher nicotine use at wave 1 was linked to increased anxiety sensitivity at wave 2. Both alcohol use and binge drinking at wave 2 were linked to increased hopelessness at wave 3. Higher nicotine use at wave 1 was linked to increased hopelessness at wave 2. Conversely, hopelessness at wave 2 was linked to decreased binge drinking at wave 3. Higher alcohol use at waves 1 and 3 was linked to increased sensation seeking at the following waves. Binge drinking at waves 2 and 3 was linked to increased sensation seeking at the following waves. Higher nicotine use at wave 2 was linked to increased sensation seeking at wave 3. In return, higher sensation seeking at wave 1 was linked to increased alcohol use at wave 2. Sensation seeking at waves 2 and 3 was linked to increased alcohol use, binge drinking, and nicotine use at the following waves. Higher alcohol and nicotine use at wave 1 were linked to increased impulsivity at the following wave. Higher alcohol and nicotine use, and binge drinking at wave 3 were linked to increased impulsivity at wave 4. In return, impulsivity at waves 1, 2 and 3 was linked to increased alcohol use, binge drinking, and nicotine use at the following waves, respectively. There were no significant findings across other waves.
Mathijssen et al. (2021)	Netherlands	*N* *=* 1121 adolescents (*M* *=* 13 years at wave 1)	Longitudinal, 3 waves across 1.5 years	Nicotine	Smoking status	Impulsivity, sensation seeking, anxiety sensitivity, hopelessness	Over the study period, initiation of nicotine use (conventional cigarettes) was linked to increases in anxiety sensitivity, impulsivity, and sensation seeking but not hopelessness.
Östlund et al. (2007)	Sweden	*N* *=* 641 female adults (from ages 30 to 70 at wave 1)	Longitudinal, 2 waves across 5 years	Alcohol	AUD	Anxiety, extraversion/introversion, conformity/non-conformity, aggression	Women who developed alcohol dependence/abuse over the study period increased in one facet of extraversion and aggression, respectively. Women who met criteria for alcohol dependence/abuse at wave 1 but not anymore at wave 2, normalized in 2 facets of anxiety, and one facet of extraversion/introversion, conformity/non-conformity, and aggression, respectively. There were no significant findings for any other personality facets.
Quinn and Harden (2013)	United States	*N* *=* 5632 adolescents (15 years at wave 1)^n^	Longitudinal, 6 waves across 10 years (at ages 15, 17, 19, 21, 23, 25)	Alcohol, nicotine, cannabis	Frequency of SU^b^	Impulsivity, sensation seeking	^d^Over the study period, change in alcohol use was positively linked to change in impulsivity and sensation seeking. Higher initial levels of alcohol use were not significantly linked to changes in impulsivity and sensation seeking, but higher levels of sensation seeking were linked to increases in alcohol use. Over the study period, change in cannabis use was positively linked to change in impulsivity but not sensation seeking. Higher initial levels of cannabis use were not significantly linked to changes in impulsivity and sensation seeking, but higher levels of sensation seeking were linked to higher increases in cannabis use. Over the study period, change in nicotine use was positively linked to change in impulsivity but not sensation seeking. Higher initial levels of nicotine use were not significantly linked to changes in impulsivity and sensation seeking, but higher levels of impulsivity were linked to increases in nicotine use.
Quinn et al. (2011)	United States	*N* *=* 1434 high school graduates (17–19 years at wave 1)	Longitudinal, 3 waves across 4 years (high school through senior year of college)	Alcohol	Heavy drinking	Impulsivity, sensation seeking, autonomy	^d^Higher levels of heavy drinking at wave 1 were linked to increases in impulsivity and sensation seeking over the study period. Likewise, higher levels of impulsivity and sensation seeking at wave 1 were linked to increases in heavy drinking over the study period. For autonomy, higher levels of autonomy were linked to lower increases in heavy drinking, but higher levels of heavy drinking showed no association with change in autonomy.
Riley et al. (2018)	United States	*N* *=* 458 college students (*M* *=* 19 years at wave 1)	Longitudinal, 3 waves across 3 years (at ages 18, 19, and 20)	Alcohol	Drinking-problems, drinking frequency	Impulsivity (i.e. urgency, lack of planning, lack of perseverance)	Within-person increases in drinking problems were linked to within-person increases in urgency, lack of planning, and lack of perseverance, over the entire study period. Within-person increases in drinking frequency were linked to within-person decreases in lack of planning and lack of perseverance over the entire study period, and in urgency only from wave 1 to 2.
Robert et al. (2020)	Germany, United Kingdom, Ireland, France	*N* *=* 726 adolescents (*M* *=* 14 years at wave 1)	Longitudinal, 3 waves across 5 years (at age 14, 16, and 19)	Alcohol	Frequency of drunkenness	Extraversion, neuroticism, openness, conscientiousness, agreeableness, sensation seeking, impulsivity, anxiety sensitivity, negative thinking	^d^Over the study period, increases in openness and impulsivity were linked to increases in drunkenness frequency. Higher impulsivity at wave 1 was linked to increases in drunkenness frequency over the study period but there was no such link for openness. Findings for the other personality traits were not significant/not reported.
Roberts and Bogg (2004)	United States	*N* *=* 99 female adults (21 years at wave 1)	Longitudinal, 3 waves across 30 years (at ages 21, 43, and 52)	Alcohol, nicotine, cannabis	Frequency of SU^b^	Social responsibility	^d^Higher cannabis use at age 43 was linked to decreases in social responsibility from age 43 to 52. There were no such significant associations between alcohol and tobacco use and changes in social responsibility. Higher levels of nicotine and cannabis use, but not alcohol use, at age 43 were linked to decreases in social responsibility from ages 21 to 43. In return, higher social responsibility at 21 was linked to increased alcohol, tobacco, and cannabis use at age 43, but not from age 43 to 52.
Samek et al. (2018)	United States	*N* *=* 2769 adolescents (17 years at wave 1)^c^	Longitudinal, 3 waves across 12 years (at ages 17, 24, and 29)	Alcohol	AUD	Constraint, negative emotionality, aggressive undercontrol	^d^Higher levels of constraint at age 17 were linked to decreased AUD symptoms at age 24, whereas AUD symptoms at age 17 showed no significant association with constraint at age 24. From ages 24 to 29, there was a negative link in both directions. There were no significant associations between negative emotionality and AUD symptoms in either direction, neither from ages 17 to 24 nor from ages 24 to 29. Higher levels of aggressive undercontrol at age 17 were linked to increased AUD symptoms at age 24, whereas AUD symptoms at age 17 showed no significant association with aggressive undercontrol at age 24. From ages 24 to 29, there was a negative link in both directions. Using biometric analysis, the authors also found that links between AUD and personality traits were largely explained by one source of shared genetic influence.
Stein et al. (1987)	United States	*N* *=* 654 young adults (21–24 years at wave 1)	Longitudinal, 2 waves across 4 years	Alcohol, cannabis, hard drugs	Frequency of SU^b^	Conscientiousness, extraversion, self-esteem, social conformity	^d^In females, higher levels of alcohol and hard drug use at wave 1 were linked to increases in one facet of self-esteem at wave 2. In return, higher levels of conscientiousness, extraversion, self-esteem and social conformity were linked to increased alcohol and cannabis use at wave 2. There were no significant associations with changes in any other personality traits, and no significant associations between cannabis use and changes in any other personality traits.
Stephan et al. (2019)	United States, Japan	*N* *=* *15 572* adults (from 5 cohort studies with nationally representative samples, *M* *=* 57 years at wave 1, s.d. *=* 6.68)^efhj^	Longitudinal, 2–3 waves across varying time periods i.e., 4 to 20 years	Nicotine	Smoking status	Extraversion, neuroticism, openness, conscientiousness, agreeableness	Over the study period, persistent smokers increased in neuroticism and decreased in openness, agreeableness, extraversion, and conscientiousness compared to non-smokers and former smokers. For extraversion and neuroticism, these findings were most consistent across cohort studies. Smoking cessation was associated with decreases in agreeableness in one study but not significantly associated with any other personality changes. Smoking at wave 1 was also linked to changes in neuroticism, openness, and agreeableness, however, these findings were little consistent across studies and directions were not reported.
Turiano et al. (2012)	United States	*N* *=* 4660 adults from a representative sample (*M* *=* 55 years at wave 2, s.d. = 12.5)^f^	Longitudinal, 2 waves across *M* *=* 9 years	Alcohol, nicotine, other drugs	Frequency of smoking behavior, frequency and volume of drinking, problem drinking behavior, illegal drug use, prescription drug use^b^	Extraversion, neuroticism, openness, conscientiousness, agreeableness	Four groups of nicotine users were identified: never, constant, new, former. There were no significant associations between nicotine user group and changes in any personality traits over the study period. Over the study period, increases in neuroticism and openness were linked to higher odds of alcohol use and problem drinking. Increases in agreeableness were linked to lower odds of alcohol use. Over the study period, increases in openness were linked to higher odds of illegal drug use, whereas increases in conscientiousness were linked to lower odds of illegal drug use. There were no significant associations with changes in any other personality traits.
Welch and Poulton (2009)	New Zealand	*N* *=* 862 young adults (18 years at wave 1)	Longitudinal, 3 waves across 4 years (at ages 18, 26, and 32)	Nicotine	Smoking status, nicotine use disorder	Constraint, negative emotionality, positive emotionality	Four groups of nicotine users were identified: nonsmoking, stopped, started, persistent. From ages 18 to 26, individuals who stopped smoking exhibited greater decreases in negative emotionality and greater increases in constraint compared to all other groups. There were no significant associations between nicotine user groups and changes in positive emotionality.
White et al. (2011)	United States	*N* *=* 503 children (*M* *=* 7 years at wave 1)	Longitudinal, 14 waves across 18 years (from age 7 to 24/25)	Alcohol	Heavy drinking, AUD (at ages 18 and 24)	Impulsive behavior	Over the study period, four impulsive behavior trajectories were identified: low, early adolescence-limited, moderate, high. Heavy drinking was linked to increases in impulsive behavior only among those with a moderate trajectory of impulsive behavior. Due to small group sizes, associations between trajectory groups and occurrence of AUD were not interpreted.
Wright and Jackson (2022)	United States	*N* = 8303 adolescents (*M* = 17 years)^n^	Longitudinal, up to 7 waves across 14 years (from age 10/11 to 24/25)	Alcohol, nicotine, cannabis, cocaine, hallucinogens, inhalants, downers (e.g. sedatives)	SU status, initiation of SU^b^	Impulsivity, sensation seeking, self-esteem	Linear and quadratic trajectories of personality traits between users and non-users were compared: Over the study period, users of nicotine, cocaine, hallucinogens, cannabis, inhalants, and downers exhibited initially increases then decreases in impulsivity compared to non-users who only exhibited decreases. Over the study period, users of general substances, nicotine, cocaine, and downers exhibited steeper increases followed by steeper decreases in sensation seeking compared to non-users who exhibited less changes overall. Over the study period, users of nicotine, cocaine, inhalants, and downers exhibited less consistent increases in self-esteem compared to non-users. There were no significant findings for users v. non-users of other substances and personality trait trajectories. Personality changes prior, during, and after SU initiation were modeled: Initiation of general SU, alcohol, nicotine, cannabis, and inhalants was associated with changes in impulsivity. Initiation of general SU, alcohol, nicotine, cocaine, and cannabis was associated with changes in sensation seeking. There were no significant associations between SU initiation of other substances and changes in impulsivity or sensation seeking, and no significant findings between SU initiation and changes in self-esteem.

SU(D), substance use (disorder); AUD, alcohol use disorder.

a Studies used data from the Household, Income, and Labour Dynamics Study (Australia).

b Analyses were run separately for substances.

c Studies used data from the Minnesota Twin Family Study (United States).

d Bidirectionality between SU and personality trait (change) was explicitly modeled.

e Studies used data from the Health and Retirement Study (HRS) (United States).

f Studies used data from the Midlife in the US Study (MIDUS) (United States).

g Studies used data from the Socio-economic Panel (GSOEP) (Germany).

h Studies used data from the Wisconsin Longitudinal Study (WLS) (United States).

i Study's primary aim was to investigate stability (not change) of personality traits.

j Studies used data from the Midlife in Japan Study (MIDJA) (Japan).

k The same sample and measure for impulsivity was used, time frames were partly overlapping.

l Analyses were run combinedly for substances.

m The same sample across a similar time frame was used but different measures for impulsivity and extraversion were employed.

n Studies used data from the National Longitudinal Study of Youth (NLSY).

**Table 2.** Intervention studies

Author year	Country	Sample	Study design	Substance use	Substance use measure	Personality construct	Key finding
Aklin et al. (2009)	United States	*N* *=* 81 patients in residential SU clinic (18–56 years, *M* *=* 38, s.d. = 10.3)	Non-randomized non-controlled intervention study, two measurement occasions: pre to post (30 days), treatment: group sessions combining elements from alcoholics and narcotics anonymous, relapse prevention, and functional analysis	Alcohol, nicotine, cannabis, ecstasy, cocaine, stimulants, sedatives, opiates, hallucinogens, phenylcyclohexyl piperidine, inhalants	Drug Use Questionnaire (DUQ)	Impulsiveness, risk taking propensity, preference for rewards (representing impulsivity)	Risk taking propensity decreased from pre- to post-treatment. There were no significant changes in impulsivity or impulsiveness.
Bayır and Aylaz (2021)	Turkey	*N* = 112 patients in a psychiatric clinic (18–44 + years, s.d. *not reported*)	Randomized controlled intervention study, two measurement occasions: pre to post (3–4 weeks) treatment: mindfulness-based psycho-education program compared with regular treatment in a different clinic	*Any*	SUD according to DSM	Self-efficacy	Self-efficacy increased from pre-to post treatment in the experimental but not in the control group.
Blonigen and Macia (2021)	United States	*N* *=* 200 military veterans in a residential SUD treatment program (25–77 years, *M* = 50, s.d. = 9.0)	Non-randomized non-controlled intervention study, three measurement occasions: admission, discharge (90–180 days) and follow-up (12-months), treatment: abstinence-based, individual- and group therapy based on cognitive behavioral therapy and 12-step facilitation approaches	Alcohol, *patient-specific drug being most problematic*	Structured Clinical Interview for Axis I disorders (SCID)	Positive emotionality, negative emotionality, constraint	Positive emotionality increased during treatment but was not sustained in the follow-up period. Negative emotionality and constraint decreased during treatment and were sustained in the follow-up period.
Borman et al. (2006)	Canada	*N* *=* 29 women in a residential SUD treatment program (18 + years, *M* *=* 38, s.d. *=* 11.0)	Non-randomized non-controlled intervention study, two measurement occasions: admission, follow-up (6 months), treatment (4 weeks): individual- and group sessions based on a holistic approach focusing on physical, emotional, social, and spiritual factors	*Any*	SUD according to DSM-IV	Extraversion, neuroticism, openness, conscientiousness, agreeableness, novelty seeking harm avoidance, reward dependence, persistence, self-directedness, cooperativeness, self-transcendence	Neuroticism and harm avoidance decreased from admission to follow-up. Self-directedness and reward dependence increased from admission to follow-up. There were no significant changes in extraversion, openness, agreeableness, and conscientiousness, novelty seeking, persistence, cooperativeness, and self-transcendence.
Carter et al. (2001)	United States	*N* *=* 230 patients from an ambulatory drug abuse treatment research clinic (*M* *=* 37 years, s.d. *not reported*)	Clinical trial (*not specified*), two measurement occasions: admission to follow-up (19 weeks), three trials: (a) opioid agonist medications (e.g. methadone), (b) behavioral incentives, and (c) opioid agonist medications and behavioral interventions	Opioid	Structured Clinical Interview for DSM-III-R (SCID-III-R)	Extraversion, neuroticism, openness, conscientiousness, agreeableness	^a^Retest-stability for all traits ranged from 0.68 to 0.74 and did not differ between drug-positive and drug-negative subjects. Neuroticism decreased, whereas extraversion and conscientiousness increased from admission to follow-up. There were no significant changes in agreeableness and openness.
Chodkiewicz and Gruszczyńska (2019)	Poland	*N* = 60 patients in an alcohol addiction treatment center (*M* = 45 years, s.d. = 10.1)	Non-randomized non-controlled intervention study, two measurement occasions: admission to completion of therapy (six weeks), treatment: multimodal CBT-based interventions (e.g. psychoeducation, skill training, 12-step program)	Alcohol	AUD according to ICD-10	Self-efficacy	There were no significant changes in self-efficacy over the study period.
Can Gür and Okanli (2019)	Turkey	*N* = 41 participants of Alcoholics Anonymous (*M* = 46 years, s.d. = 11.1)	Non-randomized controlled intervention study, four measurement occasions: pre, post (six months), four months follow-up, and six months follow-up treatment: cognitive-behavioral model-based (i.e. material incentives, aerobic exercise, and group-based behavioral training) compared to alcoholics anonymous meetings only	Alcohol	SUD according to the diagnostic criteria in DSM-5	Self-efficacy	Over the study period, self-efficacy increased in the experimental but not in the control group. However, levels of self-efficacy were higher in the experimental group v. control group only at post intervention, but not at 4-month and 6-month follow-up.
Gonçalves et al. (2014)	Brazil	*N* *=* 46 patients in a psychiatric clinic (18–45 years, *M* = 32 years, s.d. = 6.6)	Controlled intervention study, two measurement occasions: pre to post intervention (30 days), treatment: four-week standard inpatient program based on motivational interviewing and motivational chess compared with an active control group	Cocaine	Recent use and abstinence of cocaine with urine toxicology screening measure	Impulsivity	Over the study period, there was no significant change in impulsivity in either group.
Kayaoğlu and Şahin Altun (2022)	Turkey	*N* = 62 patients in a psychiatric hospital	Randomized controlled study, four measurement occasions: pre, post (three weeks), treatment: music intervention and cognitive-behavioral psychoeducation compared to routine practices of the clinic	*Any*	SUD according to DSM-V	Self-efficacy	The treatment group showed higher levels of self-efficacy compared to the control group at post-test, four and six months follow-up.
Kazemi et al. (2014)		*N* *=* 583 university students (*M* *=* 18 years, s.d. = 0.5)	Non-randomized non-controlled intervention study, three measurement occasions: baseline, three months, six months, intervention (baseline, two weeks, plus booster sessions after three months and six months): motivational interviewing, (a) mandated students and (b) voluntary students	Alcohol	Daily Drinking Questionnaire (DDQ)	Introversion/hopelessness, anxiety sensitivity, sensation seeking, impulsivity	At six months, anxiety sensitivity, impulsivity, and introversion/hopelessness were lower than at three months and at baseline. No significant changes in sensation seeking were reported over time.
Littlefield et al. (2015)	United States	*N* *=* 43 patients in a residential SU program (*M* = 36 years, s.d. *not reported*)	Non-randomized non-controlled intervention study, two measurement occasions: from admission to follow-up (*M* = 28 days later), treatment: six-week residential 12-step program	*Any*	SUD using the Mini-International Neuropsychiatric Interview (M.I.N.I.) for SUD according to DSM-IV	Impulsivity (i.e. 7 facets: delay discounting. lack of perseverance, lack of planning, negative urgency, positive urgency, sensation seeking, inhibitory control)	Over the study period, negative urgency and lack of planning decreased, whereas inhibitory control increased. There were no significant changes in delay discounting, lack of perseverance, positive urgency, and sensation seeking.
Nurco et al. (1995)	United States	*N* *=* 28 patients, minimum 6 months in methadone treatment clinic (*M* = 38 years, s.d. *not reported*)	Randomized-controlled intervention study, varying number of measurement occasions, every 3 months (*M* = 15 months), treatment: (a) clinically guided self-help program plus standard treatment (i.e. methadone maintenance and individual counseling); (b) didactic lecture program and standard treatment (control group I); (c) standard treatment (control group II)	Heroin (or similar)	*SUD* (*admitted to clinic*)	Locus of control (regarding drug misuse, externally v. internally)	In the treatment group, patients changed from external to internal locus of control, but not in the two control groups.
Oei and Jackson (1980)	Australia	*N* *=* 32 patients in a residential alcohol treatment program (*M* = 34 years, s.d. *not reported*)	Matched-controlled intervention study, four measurement occasions (pre, post, and 3-, 6-, and 12-months follow-up), treatment: (a) group social skills training; (b) individual social skills training; (c) group traditional supportive therapy; (d) individual support therapy	Alcohol	*SUD* (*admitted to clinic*), alcohol-intake inventory (i.e. amount of alcohol use over the last week)	Extraversion, neuroticism, psychoticism	Over the study period, extraversion increased, while neuroticism decreased. There was no significant change in psychoticism over time. Patients in the social skills training groups showed larger increases in extraversion, and larger decreases in psychoticism and neuroticism than patients in the support therapy groups.
Piedmont (2001)	United States	*N* *=* 99 patients in a drug rehabilitation program (23–52 years, *M* = 35, s.d. *not reported*)	Non-randomized non-controlled intervention study, three measurement occasions: pre, post (six weeks), follow-up (15 months), treatment: six-week broad multimodal intervention (incl. vocational skills training, group counseling, therapeutic activities)	*Any* (i.e. alcohol, cocaine, heroin)	Brief Symptom Inventory (BSI) Derogatis Psychiatric Rating Form (DPRS)	Extraversion, neuroticism, openness, conscientiousness,agreeableness	From pre- to post treatment, extraversion, openness, agreeableness, and conscientiousness increased, whereas neuroticism decreased. For neuroticism, agreeableness, and conscientiousness these changes were maintained at follow-up.
Stieger et al. (2022)	United States	*N* *=* 97 patients in a residential treatment center (18–29 years, *M* = 25 years, s.d. = 2.7)	Randomized intervention study, 15 measurement occasions across 28 weeks (i.e. every six weeks), treatment (four weeks): cognitive behavioral treatment and 12-step approach plus (a) mindfulness-based relapse prevention treatment, (b) social support treatment	*Any* (i.e. alcohol, cannabis, illicit drugs)	*SUD* (*admitted to center*), Substance Frequency Scale (SFS)	Conscientiousness, honesty, agreeableness, resilience, extraversion, originality	Over the study period, there was an effect of phase (treatment v. post-treatment) indicating increases in conscientiousness, honesty, resilience, extraversion, and originality during treatment. There was also an effect of time indicating increases in conscientiousness, honesty, and resilience over the study period, but this was more pronounced during the treatment phase. For agreeableness, there was only an effect of time × phase, indicating slight increases during v. post treatment but not maintained until follow-up.
Winkleby et al. (2001)	United States	*N* = 116 adolescents (9th and 10th grade, *M* = 15 years, s.d. *not reported*)	Non-randomized non-controlled intervention study, two measurement occasions: pre- to post-intervention (9 months) intervention: engaging students in an advocacy program based on social-cognitive theory	Alcohol, nicotine, cannabis	SU over the last 30 days (i.e. alcohol, nicotine, cannabis)	Self-efficacy	From pre- to posttest, self-efficacy increased in girls but not boys.
Winkleby et al. (2004)	United States	*N* = 798 students from 10 schools (*M* = 17 years, s.d. = 0.2)	Randomized-controlled intervention study, three measurement occasions: pre, post-intervention (one semester), and six-month follow-up, intervention: engaging students in an advocacy program based on social-cognitive theory compared to an existing SU prevention program (conducted in five schools, respectively)	Nicotine	Nicotine use over the last 30 days	Self-efficacy	Over the study period, the treatment group exhibited increases in self-efficacy compared to the control group, showing no significant changes.

AUD, alcohol use disorder; DSM, Diagnostic and Statistical Manual of Mental Disorders; SU(D), substance use (disorder).

a Study's primary aim was to investigate stability (not change) of personality traits.

### Observational studies

#### Samples

The sample sizes of the population-based studies varied significantly from 99 to 56 786 participants. Thirteen studies focused on adolescents, 17 on young adults (mostly university students), one on middle-aged adults only, and 7 on the entire adult lifespan.

Notably, 18 studies used at least partially overlapping data, which need to be considered when interpreting results. Specifically, Allen, Vella, & Laborde (2015), Hakulinen and Jokela (2019), and Jokela, Airaksinen, Kivimäki, and Hakulinen (2018) used data from the Household, Income, and Labour Dynamics Study (HILDA) in Australia. Jokela et al. (2018), Hakulinen and Jokela (2019), Luchetti, Terracciano, Stephan, and Sutin (2018), and Stephan, Sutin, Luchetti, Caille, and Terracciano (2019) used data from the Health and Retirement Study (HRS) in the US. Jokela et al. (2018) and Stephan et al. (2019) used data from the Midlife in Japan Study (MIDJA). Jokela et al. (2018), Hakulinen and Jokela (2019), and Turiano, Whiteman, Hampson, Roberts, and Mroczek (2012) used data from the Midlife in the US Study (MIDUS). Jokela et al. (2018) and Hakulinen and Jokela (2019) used data from the German Socio-economic Panel (GSOEP). Jokela et al. (2018), Hakulinen and Jokela (2019), and Stephan et al. (2019) used data from the Wisconsin Longitudinal Study (WLS) in the US. Blonigen et al. (2015), Hicks, Durbin, Blonigen, Iacono, and McGue (2012), and Samek et al. (2018) used data from the Minnesota Twin Family Study in the US. Quinn and Harden (2013) and Wright and Jackson (2022) used data from the National Longitudinal Study of Youth (NLSY) in the US. Littlefield, Sher, and Wood (2009), Littlefield, Sher, and Wood (2010b), Littlefield, Sher, and Steinley (2010a), Littlefield and Sher (2012), and Littlefield, Vergés, Wood, and Sher (2012) used data from the same student sample with similar time frames but considered different measures of impulsivity and extraversion. Kaiser, Bonsu, Charnigo, Milich, and Lynam (2016) and Kaiser, Davis, Milich, Smith, and Charnigo (2018) used data from the same sample and measure of impulsivity; time frames were partly overlapping.

#### Study designs

In line with our search criteria, all studies were based on a longitudinal design, with number of waves ranging from 2 to 14 (median: 3 waves). The time span over which personality changes were assessed varied between 17 months and 30 years (median: 5 years). We present standardized effect sizes as exemplary but refrain from interpretations due to the large heterogeneity of studies.

#### Substances

In the observational studies, 31 studies investigated alcohol, 12 nicotine, 9 cannabis, and one ecstasy use. In addition, 4 studies investigated multiple drug use including cocaine and sedatives, and 2 examined SU in general without differentiation between different substances. Indicators of both SU and personality are described in online Supplementary Table S2.)

#### Extraversion-related traits (18 studies)

Seven of 15 studies reported at least one significant association between alcohol use and changes in extraversion (Hakulinen & Jokela, 2019; Jokela et al., 2018; Klimstra et al., 2014; Luchetti et al., 2018; Östlund, Hensing, Sundh, & Spak, 2007), positive emotionality (Blonigen et al., 2015), or sociability (Gmel, Marmet, Studer, & Wicki, 2020). However, these findings were inconsistent and varied, for example, by facet of extraversion (Östlund et al., 2007), measure, and cohort (Hakulinen & Jokela, 2019). Eight studies in adults of different ages found no significant associations between alcohol use and extraversion-related changes using various SU(D) measures (Allen et al., 2015; Hicks et al., 2012; Kroencke et al., 2021; Littlefield et al., 2009, 2010b; Robert et al., 2020; Stein, Newcomb, & Bentler, 1987; Turiano et al., 2012).

For nicotine use, research found that initiation of smoking was linked to a small increase in extraversion (*β* = 0.07) (Jokela et al., 2018) or that persistent smokers became less extraverted (*d* = 0.09–0.22) (Stephan et al., 2019). Other studies found that nicotine use was unrelated to changes in extraversion (Kroencke et al., 2021; Turiano et al., 2012) and positive emotionality (Welch & Poulton, 2009).

For cannabis and other drug use, one study found that higher cannabis use was associated with increases in extraversion in university students (Klimstra et al., 2014), while 2 studies found no significant links in adult samples (Kroencke et al., 2021; Turiano et al., 2012). Taken together, the evidence on alcohol, nicotine, and other SU and changes in extraversion was inconsistent, with different directions of change, depending on age and personality measure.

#### Neuroticism-related traits (21 studies)

Eleven of 16 studies found that alcohol use was associated with increases in at least one neuroticism-related trait in samples of all ages (Allen et al., 2015; Blonigen et al., 2015; Gmel et al., 2020; Hakulinen & Jokela, 2019; Hicks et al., 2012; Littlefield et al., 2009, 2010b; Malmberg et al., 2013; Östlund et al., 2007; Samek et al., 2018; Turiano et al., 2012), although these findings were small (e.g. *β* = 0.04 in Allen et al., 2015) and varied by trait: Increased alcohol use was associated with increases in aggressive undercontrol but not negative emotionality in Samek et al. (2018), increases in aggression hostility but not neuroticism-anxiety in Gmel at al. (2020), and increases in hopelessness but not anxiety sensitivity in Malmberg et al. (2013). Five studies found no significant associations between alcohol use and neuroticism-related traits (Jokela et al., 2018; Klimstra et al., 2014; Kroencke et al., 2021; Luchetti et al., 2018; Robert et al., 2020).

Six of 10 studies found that nicotine use was linked to increases in neuroticism-related traits (Allen et al., 2015; Littlefield & Sher, 2012; Malmberg et al., 2013; Mathijssen, Rozema, Hiemstra, Jansen, & van Oers, 2021; Stephan et al., 2019; Welch & Poulton, 2009), although these effects were small (e.g. *β* = 0.07 for hopelessness in Malmberg et al., 2013) and often not consistent over the study period (e.g. Littlefield & Sher, 2012; Malmberg et al., 2013). One study found a small decrease in neuroticism following smoking initiation (Kroencke et al., 2021), and 3 studies found no significant association between nicotine use and neuroticism (Jokela et al., 2018; Turiano et al., 2012) or hopelessness (Mathijssen et al., 2021).

For cannabis and other drugs, one study found that increasing sedative use was associated with subsequent increases in neuroticism (Kroencke et al., 2021), while 2 studies found no significant associations (Klimstra et al., 2014; Turiano et al., 2012). In sum, this indicates an association between higher alcohol use and increases in some neuroticism-related traits, while the evidence is less consistent for nicotine and other substances.

#### Openness-related traits (11 studies)

Four studies found associations between higher alcohol use and increases in openness (Klimstra et al., 2014; Robert et al., 2020; Turiano et al., 2012) or novelty seeking (Littlefield et al., 2012), mostly in young adults. In contrast, 5 studies (mostly based on adults of all ages) found no such associations (Allen et al., 2015; Hakulinen & Jokela, 2019; Jokela et al., 2018; Littlefield et al., 2010b; Luchetti et al., 2018).

For nicotine use, one study found that openness decreased among persistent smokers (Stephan et al., 2019), while 4 studies found no significant association between nicotine use and change in openness (Allen et al., 2015; Jokela et al., 2018; Kroencke et al., 2021; Turiano et al., 2012). For cannabis and other drug use, one study found that higher cannabis was linked to increases in openness and its facet unconventionality in a college sample (Klimstra et al., 2014), while other research found no significant associations in adults (Kroencke et al., 2021). Overall, some evidence suggests that alcohol and cannabis use may be associated with increases in openness in adolescents and young adults.

#### Conscientiousness-related traits (16 studies)

Eight of 14 studies found that alcohol use was associated with decreases in conscientiousness-related traits (Blonigen et al., 2015; Hakulinen & Jokela, 2019; Hicks et al., 2012; Jokela et al., 2018; Klimstra et al., 2014; Littlefield et al., 2010b; Luchetti et al., 2018; Samek et al., 2018), with small effect sizes (e.g. *β* = 0.09 in Jokela et al., 2018). Six studies found no significant associations (Allen et al., 2015; Kroencke et al., 2021; Robert et al., 2020; Roberts & Bogg, 2004; Stein et al., 1987; Turiano et al., 2012), potentially because they mostly relied on frequency measures of SU (rather than problematic SU).

Four studies found that nicotine use was associated with decreases in conscientiousness, mostly in young adults (Allen et al., 2015; Roberts & Bogg, 2004; Stephan et al., 2019; Welch & Poulton, 2009), while 4 studies found no significant associations in adults of all ages (Jokela et al., 2018; Kroencke et al., 2021; Stein et al., 1987; Turiano et al., 2012). Roberts and Bogg (2004) found that cannabis use was associated with decreases in social responsibility in young women, while Kroencke et al. (2021) found no significant associations in an older sample of both genders. There were no significant associations between other drug use and changes in conscientiousness-related traits (Kroencke et al., 2021; Stein et al., 1987; Turiano et al., 2012). In summary, there is some (albeit inconsistent) evidence that SU may relate to small decreases in conscientiousness.

#### Agreeableness-related traits (13 studies)

Three studies found that higher alcohol use was associated with decreases in agreeableness (Hakulinen & Jokela, 2019; Klimstra et al., 2014; Turiano et al., 2012), and one study found that trait (non)conformity ‘normalized’ after remission from alcohol use disorder (Östlund et al., 2007). However, in 7 studies, alcohol use was unrelated to changes in agreeableness-related traits (Allen et al., 2015; Jokela et al., 2018; Kroencke et al., 2021; Littlefield et al., 2010b; Luchetti et al., 2018; Robert et al., 2020; Stein et al., 1987).

Other drug (Kroencke et al., 2021; Turiano et al., 2012) and nicotine (Allen et al., 2015) use were linked to decreases in agreeableness, and, conversely, smoking cessation to increases in agreeableness (Stephan et al., 2019). In other studies, nicotine (Jokela et al., 2018; Kroencke et al., 2021; Stein et al., 1987; Turiano et al., 2012) and cannabis (Klimstra et al., 2014; Kroencke et al., 2021) use were unrelated to changes in agreeableness. Taken together, a few studies suggest that SU(D) is associated with decreases in agreeableness-related traits, but this evidence is inconsistent.

#### Impulsivity-related traits (19 studies)

Ten of 10 studies found that alcohol use was associated with changes in impulsivity (Ashenhurst, Harden, Corbin, & Fromme, 2015; Kaiser et al., 2016; 2018; Littlefield et al., 2009; 2010a; Malmberg et al., 2013; Quinn & Harden, 2013; Riley, Davis, Milich, & Smith, 2018; White et al., 2011; Wright & Jackson, 2022), and 6 of 6 studies (based on adolescent and young adult samples) found at least one association with changes in sensation seeking (Ashenhurst et al., 2015; Kaiser et al., 2016; Kaiser et al., 2018; Malmberg et al., 2013; Quinn & Harden, 2013; Wright & Jackson, 2022). The direction of change was almost exclusively positive and small to moderate in size. For instance, a within-person increase in drinking problems were linked to increases in multiple facets of impulsivity (e.g. lack of planning, *β* = 0.18–0.20) among college students (Riley et al., 2018). However, some studies only found associations between individual waves (Littlefield et al., 2010a) or for specific SU measures (Malmberg et al., 2013).

Five of 5 studies found that nicotine use was associated with increases in impulsivity (Littlefield & Sher, 2012; Malmberg et al., 2013; Mathijssen et al., 2021; Quinn & Harden, 2013; Wright & Jackson, 2022), and 3 of 4 studies found that nicotine use was associated with increases in sensation seeking (Malmberg et al., 2013; Mathijssen et al., 2021; Wright & Jackson, 2022) in adolescents.

For cannabis use, Quinn and Harden (2013) found increases in impulsivity but not sensation seeking from age 15 to 25, consistent with Wright and Jackson (2022). De Win et al. (2006) found that higher ecstasy use was linked to increases in sensation seeking but not impulsivity. Wright and Jackson (2022) found that impulsivity and sensation seeking increased more strongly in adolescent cocaine users *v.* non-users. In summary, several studies suggest that SU relates to increases in impulsivity and sensation seeking, especially in adolescents and young adults.

#### Self-esteem and related traits (4 studies)

Stein et al. (1987) found that alcohol use was associated with increases in one facet of self-esteem in women, while Kroencke et al. (2021) and Wright and Jackson (2022) found no such association. Wright and Jackson (2022) found that self-esteem increased less consistently during adolescence in smokers *v.* non-smokers, whereas 2 studies found no significant association (Kroencke et al., 2021; Stein et al., 1987). Moreover, self-esteem increased less consistently in adolescent users (Wright & Jackson, 2022) and decreased in adult users (Kroencke et al., 2021) of sedatives/downers, whereas Stein et al. (1987) reported increases in one facet of self-esteem in young adults, although not differentiating between types of drugs. Taken together, previous research suggests no consistent association between SU and changes in self-esteem.

#### Other (4 studies)

Other studies investigated traits such as identity status (De Moor, Sijtsema, Weller, & Klimstra, 2022), cognitive structure (Labouvie & McGee, 1986), autonomy (Labouvie & McGee, 1986; Quinn, Stappenbeck, & Fromme, 2011), or psychosocial maturity (Chassin et al., 2010). For instance, in adolescent juvenile offenders, Chassin et al. (2010) found higher alcohol/cannabis use being associated with decreasing psychosocial maturity (*β* = −0.03/–0.05).

#### Directionality and within-person changes

Most observational studies investigating the directionality of change (online Supplementary Table S4) yielded bidirectional associations between SU and personality change. However, there was slightly more evidence for personality predicting subsequent changes in SU than for SU predicting subsequent personality changes. Studies that modeled between-person *v.* within-person effects evidenced within-person increases in impulsivity with higher/increasing SU (in college students) (Riley et al., 2018) but found little evidence for associations between SU and within-person changes in the Big Five (Jokela et al., 2018; Kroencke et al., 2021) (in adults of all ages).

### Intervention studies

#### Samples

Sample sizes in the intervention studies varied from 28 to 798. In contrast to the observational studies, the samples of the intervention studies mostly consisted of patients with (sub-)threshold SUD undergoing treatment. Only 3 studies examined non-clinical student samples (Kazemi, Levine, Dmochowski, Angbing, & Shou, 2014; Winkleby, Feighery, Altman, Kole, & Tencati, 2001, 2004).

#### Study designs

Assessment periods of the intervention studies ranged from four weeks to considerably longer timeframes up to 15 months post-intervention. Most studies were based on 3 or 4 waves and were conducted without control conditions. Only 4 studies used a randomized controlled design (RCT) (Bayır & Aylaz, 2021; Kayaoğlu & Şahin Altun, 2022; Nurco et al., 1995; Winkleby et al., 2001), one study used a randomized design (Stieger, Allemand, Roberts, & Davis, 2022), 2 studies used a non-randomized control-group design (Can Gür & Okanli, 2019; Gonçalves et al., 2014), and one study used matched control groups (Oei & Jackson, 1980).

#### Substances

Of the 17 intervention studies, 4 focused on alcohol use, one focused on nicotine use, one focused on heavy use of opioids, cocaine, and heroin, respectively, and nine were intervention studies that focused on treatment programs for heavy SU of various kinds and did not test for substance-specific effects. Indicators of SU(D) and personality that were considered in these studies are described in online Supplementary Table S2.

#### Interventions

The studies encompassed various treatments, such as individual- and group-based cognitive-behavioral therapy and 12-step facilitation approaches to recovery (adapted from Alcoholics/Narcotics Anonymous) (Aklin, Tull, Kahler, & Lejuez, 2009; Blonigen & Macia, 2021; Can Gür & Okanli, 2019; Chodkiewicz & Gruszczyńska, 2019; Kayaoğlu & Şahin Altun, 2022; Littlefield et al., 2015; Stieger et al., 2022), motivational interventions (Gonçalves et al., 2014; Kazemi et al., 2014), mindfulness-based interventions (Bayır & Aylaz, 2021; Stieger et al., 2022), but also less usual interventions such as motivational chess (Gonçalves et al., 2014), music (Kayaoğlu & Şahin Altun, 2022), or advocacy training for students (Winkleby et al., 2001; 2004). Durations of the interventions ranged from 4 to 25 weeks.

#### Extraversion-related traits (6 studies)

Two studies found that extraversion increased from admission to 19-week follow-up in patients from an opioid treatment clinic using agonist medication and behavioral interventions (Carter et al., 2001) and from pre-treatment to 12-month follow-up in patients from a residential alcohol treatment program using social skills training and supportive therapy (Oei & Jackson, 1980).

Three studies found that extraversion increased in SUD patients receiving cognitive behavioral therapy (CBT) and other multimodal interventions only from pre- to post-treatment, but not until 6-month (Stieger et al., 2022), 12-month (Blonigen & Macia, 2021), or 15-month follow-up (Piedmont, 2001). One study found no changes in extraversion in relation to SUD treatment (Borman et al., 2006). In summary, there is some evidence for increases in extraversion in SUD patients during treatment that, however, are not sustained in the long term.

#### Neuroticism-related traits (6 studies)

All studies found decreases in neuroticism-related traits in relation to different SU(D) interventions (e.g. CBT, motivation intervention program, social skills training) and settings (e.g. outpatient (Carter et al., 2001; Piedmont, 2001), inpatient (Blonigen & Macia, 2021; Borman et al., 2006; Oei & Jackson, 1980), university (Kazemi et al., 2014)). Lower levels of neuroticism-related traits were maintained up to 15 months post-treatment (*d* = 0.28 mean Big Five change) (Piedmont, 2001). Social skills training was associated with greater decreases in neuroticism than supportive therapy in alcohol use patients (Oei & Jackson, 1980). Taken together, the evidence suggests that SU(D) interventions are associated with sustained reductions in neuroticism.

#### Openness-related traits (4 studies)

Two studies found increases in openness-related traits in SUD patients from pre- to post-treatment (i.e. CBT and comprehensive multimodal interventions) that, however, were not maintained until 6-month (Stieger et al., 2022) and 15-month (Piedmont, 2001) follow-up. Conversely, no changes in openness-related traits were found in relation to multimodal (Borman et al., 2006) and opioid (Carter et al., 2001) treatment. Thus, previous evidence suggests no lasting changes in openness-related traits following SUD treatment.

#### Conscientiousness-related traits (5 studies)

Four studies found lasting increases (up to 15-month follow-up) in conscientiousness (Carter et al., 2001; Piedmont, 2001; Stieger et al., 2022) and constraint (Blonigen & Macia, 2021) in relation to (cognitive) behavioral approaches (e.g. *d* = 0.37 from pre- to post-treatment in Stieger et al., 2022). In contrast, Borman et al. (2006) found no significant changes in conscientiousness and persistence but increases in self-directedness from admission to 6-month follow-up in an intervention focusing on physical, emotional, social, and spiritual factors. Taken together, there is initial evidence that cognitive-behavioral SUD treatment relates to increases in conscientiousness-related traits.

#### Agreeableness-related traits (4 studies)

One study found increases in agreeableness in SUD patients up to 6-month follow-up (Piedmont, 2001), another found increases from pre- to post-treatment, but this effect disappeared at 6-month follow-up (Stieger et al., 2022). Two studies found no significant changes in agreeableness-related traits related to SUD treatment (Borman et al., 2006; Carter et al., 2001). The evidence provides little support for lasting increases in agreeableness following SUD treatment.

#### Impulsivity-related traits (4 studies)

Kazemi et al. (2014) found a decrease in impulsivity among university students who participated in an alcohol intervention to motivate change in drinking behavior (either mandated or voluntarily) from baseline to 6-month follow-up, including booster sessions. In inpatient multimodal SU treatments, Littlefield et al. (2015) found decreases in some facets of impulsivity, while Aklin et al. (2009) found no significant changes in impulsivity but decreases in risk taking, both after 4 weeks of intervention. Gonçalves et al. (2014) found no changes in impulsivity following a 4-week motivational interviewing and chess intervention for cocaine users in a psychiatric clinic. For sensation seeking, no significant changes in relation to SUD treatment were found (Kazemi et al., 2014; Littlefield et al., 2015). These results provide some, but inconsistent evidence for decreases in impulsivity-related traits in relation to SU(D) interventions.

#### Self-efficacy and related traits (7 studies)

Only studies that focus on general (but not domain-specific) self-efficacy are considered, as the focus of this review is on major personality traits. RCTs in SUD patients found that clinically guided self-help plus standard treatment (i.e. methadone maintenance and counselling) (Nurco et al., 1995), mindfulness-based psychoeducation (Bayır & Aylaz, 2021), CBT with music intervention (Kayaoğlu & Şahin Altun, 2022), and advocacy training to reduce smoking in university students (Winkleby et al., 2004) led to increases in self-efficacy (or changes from external to internal locus of control, Nurco et al., 1995). Two studies without a randomized controlled design also found that self-efficacy increased in university students following an advocacy intervention to reduce smoking (Winkleby et al., 2001) and in alcohol use patients from pre- to post-treatment (i.e. CBT plus exercise incentives) but not to 4- or 6-month follow-up (Can Gür & Okanli, 2019). One study found no changes in self-efficacy after 6-week CBT (Chodkiewicz & Gruszczyńska, 2019). Taken together, these findings suggest that most SU(D) interventions increase self-efficacy.

#### Other (3 studies)

Other personality-like traits investigated in the context of SU(D) interventions were reward dependence (Borman et al., 2006), psychoticism (Oei & Jackson, 1980), or resilience (Stieger et al., 2022). For instance, Stieger et al. (2022) found that SUD patients exhibited increases in trait resilience up to 6 months after CBT.

## Discussion

This systematic review synthesized existing evidence on personality changes associated with SU(D). In observational studies, higher or increasing SU was most consistently linked to increases in impulsivity, sensation seeking (to a lesser extent), and less consistently neuroticism and related traits. SU(D) interventions were linked to decreases in neuroticism, which is consistent with meta-analytic findings that psychological interventions were related to reductions in neuroticism (Roberts et al., 2017). For impulsivity and sensation seeking, there was only weak support for intervention-related changes. However, intervention studies provided support for initial increases in self-efficacy and sustained increases in conscientiousness with treatment/amelioration of SU(D), whereas observational studies provided no consistent evidence for changes in self-esteem and suggested that only severe SU may be linked to decreases in conscientiousness. For traits related to extraversion, agreeableness, and openness, findings were inconsistent and rather weak in both types of studies, as discussed below.

Differences in findings might be due to differences in personality and SU measures, samples, and study designs, often referring to different age groups (see online Supplementary Table S5). Some studies suggest that developmental factors play an important role, such that certain SU-related personality changes especially occur in younger individuals. For example, positive associations between alcohol/cannabis use and openness-related traits were restricted to younger individuals, and nicotine use was associated with increased impulsivity in adolescents but not in young adults, consistent with findings that differences in impulsivity between non-clinical adolescent users and non-users did not persist into adulthood (Wright & Jackson, 2022). Furthermore, the personality trait, the severity of SU(D), and the context are important. For example, alcohol use has been associated with higher levels of extraversion particularly in the college context (Alexander, Howard, & Maggs, 2022), but it is possible that increases in extraversion are only found when considering the frequency of (binge) drinking (e.g. at student parties) (Klimstra et al., 2014) rather than negative consequences and SUD symptoms (Littlefield et al., 2009, 2010b). Conversely, the evidence for a negative link between SU and changes in conscientiousness-related traits is most consistent when studies examine symptoms of SUD (e.g. Hicks et al. 2012; Littlefield et al. 2010b; Samek et al. 2018) rather than just frequency of SU. We also identified a gap in research: observational studies in adolescents have almost exclusively investigated changes in impulsivity and sensation seeking, whereas studies in (middle-aged) adults have almost exclusively examined change in the Big Five. Thus, future research in the context of SU may additionally focus on changes in the Big Five during adolescence.

Regarding bidirectional associations between SU and personality, observational studies provided slightly more evidence for personality predicting changes in SU (*vulnerability model*) than for SU predicting changes in personality (*scar model*), but these effects were rarely directly compared. Consistent with the *transactional model*, almost all studies reported (at least some) associations in both directions, highlighting that SU and personality are closely intertwined: Certain personality trait levels (e.g. high impulsivity) predispose to increased SU (selection effects). At the same time, increasing SU accentuates these trait levels over time, leading to a vicious cycle of SU and associated (personality) problems (socialization effects).

Intervention studies found that neuroticism decreased during SU(D) treatment, while other personality changes varied by type of intervention. Extraversion increased with social skills training, while conscientiousness, agreeableness, and self-efficacy increased with cognitive-behavioral interventions that focused on stress coping and regaining control over SU. Consistent with this idea, Littlefield et al. (2010b) found that more functional coping mediated the association between decreases in SU and increases in conscientiousness. Personality changes were often not maintained until follow-up, highlighting the need for additional booster sessions after treatment. However, only 4 studies were based on randomized controlled designs, so there is limited causal evidence that SU(D) interventions induce personality changes.

Most observational studies focused on the frequency and intensity of SU without assessing diagnostic criteria for full-threshold SUD (but see Hicks et al., 2012; Östlund et al., 2007; Samek et al., 2018). Thus, they refer only to personality changes related to more (or less) frequent SU regardless of clinical features. The intervention studies mostly focused on individuals with full-threshold SUD but did not directly assess whether changes in SU(D) were related to personality changes. That is, there is little (direct) evidence on personality changes before, during, and after onset or remission of SU(D) or in relation to specific clinical features, as specified by our research questions. In general, the studies included in this review were highly heterogeneous in terms of measures, samples, study designs, and statistical approaches (see also online Supplementary Table S5).

Because individual substances (e.g. sedatives *v.* stimulants) have different physiological effects, substance-specific personality changes are plausible. At the same time, SU-related personality changes may be due to general processes associated with heavy use (e.g. craving and withdrawal) independent of a particular drug (Chen, 2022). Observational studies found that personality changes varied by substance, but rarely focused on full-threshold SUD. Intervention studies focused on SUD but rarely distinguished between substances, highlighting the need for future research in this field.

To the best of our knowledge, this review provides the first comprehensive aggregation and systematic synthesis of evidence on SU(D)-related personality changes. However, it is not without limitations: First, our focus was on clinically relevant SU(D). However, because most studies focused on SU frequency and intensity irrespective of SUD, it was virtually impossible to analyze associations with specific clinical features and diagnostic transitions (e.g. onset and remission of SUD). At the same time, this limitation could be considered a strength given the increasing importance of dimensional approaches in clinical psychology (Kotov et al., 2021; Krueger et al., 2021). Second, we only included peer-reviewed studies published in English or German, potentially limiting generalizability.

## Conclusions

In observational studies, SU(D) was most consistently related to increases in impulsivity-related traits. In intervention studies, decreases in SU(D) were linked to decreases in neuroticism-related traits and increases in conscientiousness and self-efficacy, although the available literature was sparse. Associations between SU(D) and other personality traits varied by substance and developmental stage. Overall, studies were highly heterogeneous in measures, samples, study designs, and statistical approaches. Future meta-analyses may investigate whether and how changes in specific personality traits vary by substance and different indicators of SU(D) (e.g. frequency of use *v.* clinical features). Practitioners may particularly target traits related to impulsivity, neuroticism, conscientiousness, and self-efficacy to treat but also prevent SU(D). Tailored interventions based on personality information, such as the PreVenture program (Debenham et al., 2021; Newton et al., 2022), have been shown to be effective, and could be implemented on a larger scale.

## Preregistration statement

This systematic review was preregistered in the PROSPERO Systematic Reviews Database (CRD42022370973).

## Supporting information

Juchem et al. supplementary material Juchem et al. supplementary material

## Data Availability

No datasets were generated or analyzed for the current study.

## Supplementary material

The supplementary material for this article can be found at https://doi.org/10.1017/S003329172400093X.
