# Supplementary material for: Personality changes related to presence and treatment of substance use (disorders): a systematic review
Source: Psychol Med. 2024 Apr 22;54(9):1905–29. doi: 10.1017/S003329172400093X (PMC11413350; doi:10.1017/S003329172400093X)
Supplement: Juchem et al. supplementary material [file S003329172400093Xsup001.pdf]

Supplementary material

Table S1

Systematic Review Search Terms

(
[Personality-related terms]
(Personality OR Character Trait OR Character Strength* OR Big Five OR Hexaco OR Neuroticis* OR Emotional stability OR Openness OR Extraversion OR Agreeableness OR Conscientiousness OR Honesty-humility OR Locus of Control OR Perceived control OR Control belief OR Self-efficacy OR Self-esteem OR Mental toughness OR Hardiness OR Behavioral Inhibition OR Behavioral disinhibition OR Dark triad OR Narcissis* OR Machiavellian* OR Psychopath* OR Risk avers* OR Risk affin* OR Harm avoidan* OR Novelty seek* OR Sensation seek* OR Reward dependen*)
AND
[Change-related terms]
(Change* OR Development OR Modification OR Malleab*)
)
AND
(
[Addiction-related terms]
(Addiction OR Addicted OR Dependen* OR Misuse OR Problematic Use OR Use Disorder)
AND
[Substance-related terms]
(Drug* OR Substance* OR Alcohol OR Heavy Drinking OR Cannabis OR THC OR Tetrahydrocannabinol OR Cocaine OR Amphetamine* OR Methamphetamine* OR Opia* OR Opio* OR Dissociatives OR Ketamine OR Psylocibin OR GHB OR GBL OR Gamma-Butyrolacton OR Hydroxybutyric Acid OR LSD OR Lysergic acid diethylamid* OR MDMA OR Ecstasy OR Methylamphetamin* OR Nicotine OR Heavy Smoking OR Benzodiazepin*)
)

Table S2

Indicators of substance use (disorder) (SUD) and personality change

Observational population-based studies

Indicators of substance use. SU(D) was mainly assessed via self-report. Specifically, participants were asked to indicate the frequency and quantity of SU within a specific time frame (e.g., within the past year). Response options spanned a range from never/zero to daily. The items used were often self-constructed. A few studies used items from established clinical assessment tools or short screening scales (e.g., AUDIT; Gmel et al., 2020). However, only 7 studies employed comprehensive measures capable to diagnose clinically relevant SUD based on established diagnostic criteria (Hemphälä, Kosson, Westerman, & Hodgins, 2015; Hicks, Durbin, Blonigen, Iacono, & McGue, 2012; Littlefield & Sher, 2012; Östlund, Hensing, Sundh, & Spak, 2007; Samek et al., 2018; Welch & Poulton, 2009; White et al., 2011). Thus, only a minority of population-based studies focused on personality changes in the context of full-threshold SUD according to ICD or DSM.

Indicators of personality change. Personality traits were mostly measured via self-report, except for one study with additional parental reports (White et al., 2011). In most cases, well-established scales were used such as the Neo-Five-Factor Inventory (NEO-FFI), the Zuckerman-Kuhlman Personality Questionnaire, or the Barratt-Impulsiveness Scale (BIS-11). However, in some cases, individual items were combined from different scales.

Intervention studies

Indicators of SU. SU(D) were assessed with various measures, including mostly self-report questionnaires (Aklin et al., 2009; Kazemi et al., 2014; Stieger et al., 2022) but also structured clinical interviews (Blonigen & Macia, 2021; Carter et al., 2001; Littlefield et al., 2015) and, in one study, objective physiological measures (i.e., urine toxicology screening) (Gonçalves et al., 2014).

Indicators of personality change. Personality traits were mostly assessed with self-report questionnaires (e.g., NEO-PI-R; Borman et al., 2006; Carter et al., 2001; Piedmont, 2001). However, in 2 studies, additional behavioral computer-administered tasks were conducted to assess risk-taking (e.g., Balloon Analogue Risk Task; Aklin et al., 2009), impulsivity (e.g., Delay Discounting Procedure; Aklin et al., 2009), and inhibitory control (stop-signal task STOP-IT; Littlefield et al., 2015).

Table S3a

Personality trait clusters observational studies

Cluster	Study included in review	Personality construct
**Extraversion and related traits**	Allen, Vella, and Laborde (2015)	Extraversion
	Hakulinen and Jokela (2019)	Extraversion
	Jokela et al. (2018)	Extraversion
	Klimstra, Luyckx, Hale, and Goossens (2014)	Extraversion
	Kroencke, Kuper, Bleidorn, and Denissen (2021)	Extraversion
	Littlefield, Sher, and Wood (2009)	Extraversion
	Littlefield, Sher, and Wood (2010)	Extraversion
	Luchetti, Terracciano, Stephan, and Sutin (2018)	Extraversion
	Robert et al. (2020)	Extraversion
	Stein, Newcomb, and Bentler (1987)	Extraversion
	Stephan, Sutin, Luchetti, Caille, and Terracciano (2019)	Extraversion
	Turiano, Whiteman, Hampson, Roberts, and Mroczek (2012)	Extraversion
	Östlund et al. (2007)	Extraversion/Introversion
	Gmel, Marmet, Studer, and Wicki (2020)	Sociability
	Blonigen et al. (2015)	Positive Emotionality
	Hicks et al. (2012)	Positive Emotionality
	Welch and Poulton (2009)	Positive Emotionality
	Labouvie and McGee (1986)	Play
	Labouvie and McGee (1986)	Exhibition
**Neuroticism and related traits**	Allen et al. (2015)	Neuroticism
	Hakulinen and Jokela (2019)	Neuroticism
	Jokela et al. (2018)	Neuroticism
	Klimstra et al. (2014)	Neuroticism
	Kroencke et al. (2021)	Neuroticism
	Littlefield et al. (2009)	Neuroticism
	Littlefield, Sher, and Wood (2010)	Neuroticism
	Littlefield and Sher (2012)	Neuroticism
	Luchetti et al. (2018)	Neuroticism
	Robert et al. (2020)	Neuroticism
	Stephan et al. (2019)	Neuroticism
	Turiano et al. (2012)	Neuroticism
	Bloningen et al. (2015)	Negative Emotionality
	Hicks et al. (2012)	Negative Emotionality
	Samek et al. (2018)	Negative Emotionality
	Welch and Poulton (2009)	Negative Emotionality
	Östlund et al. (2007)	Aggression
	Gmel et al. (2020)	Aggression Hostility
	Samek et al. (2018)	Aggressive Undercontrol
	Östlund et al. (2007)	Anxiety
	Malmberg et al. (2013)	Anxiety Sensitivity
	Mathijssen, Rozema, Hiemstra, Jansen, and van Oers (2021)	Anxiety Sensitivity
	Robert et al. (2020)	Anxiety Sensitivity
	Labouvie and McGee (1986)	Harm-Avoidance
	Malmberg et al. (2013)	Hopelessness
	Gmel et al. (2020)	Neuroticism-Anxiety
**Openness and related traits**	Allen et al. (2015)	Openness
	Hakulinen and Jokela (2019)	Openness
	Jokela et al. (2018)	Openness
	Klimstra et al. (2014)	Openness
	Kroencke et al. (2021)	Openness

	Littlefield, Sher, and Wood (2010)	Openness
	Luchetti et al. (2018)	Openness
	Robert et. al (2020)	Openness
	Stephan et al. (2019)	Openness
	Turiano et al. (2012)	Openness
	Robert et al. (2020)	Sensation Seeking
	Littlefield, Vergés, Wood, and Sher (2012)	Novelty Seeking
**Conscientiousness and related traits**	Allen et al. (2015)	Conscientiousness
	Hakulinen and Jokela (2019)	Conscientiousness
	Jokela et al. (2018)	Conscientiousness
	Klimstra et al. (2014)	Conscientiousness
	Kroencke et al. (2021)	Conscientiousness
	Littlefield, Sher, and Wood (2010)	Conscientiousness
	Luchetti et al. (2018)	Conscientiousness
	Robert et al. (2020)	Conscientiousness
	Stein et al. (1987)	Conscientiousness
	Stephan et al. (2019)	Conscientiousness
	Turiano et al. (2012)	Conscientiousness
	Blonigen et al. (2015)	Constraint
	Hicks et al. (2012)	Constraint
	Samek et al. (2018)	Constraint
	Welch and Poulton (2009)	Constraint
	Roberts and Bogg (2004)	Social Responsibility
	Labouvie and McGee (1986)	Achievement
**Agreeableness and related traits**	Allen et al. (2015)	Agreeableness
	Hakulinen and Jokela (2019)	Agreeableness
	Jokela et al. (2018)	Agreeableness
	Klimstra et al. (2014)	Agreeableness
	Kroencke et al. (2021)	Agreeableness
	Littlefield, Sher, and Wood (2010)	Agreeableness
	Luchetti et al. (2018)	Agreeableness
	Robert et al. (2020)	Agreeableness
	Stephan et al. (2019)	Agreeableness
	Turiano et al. (2012)	Agreeableness
	Stein et al. (1987)	Social Conformity
	Östlund et al. (2007)	Conformity/Non-Conformity
	Labouvie and McGee (1986)	Affiliation
**Self-esteem and related traits**	Kroencke et al. (2021)	Self-esteem
	Labouvie and McGee (1986)	Self-esteem
	Stein et al. (1987)	Self-esteem
	Wright and Jackson (2022)	Self-esteem
**Impulsivity and related traits**	Ashenhurst et al. (2015)	Impulsivity
	De Win et al. (2006)	Impulsivity
	Kaiser et al. (2016)	Impulsivity
	Kaiser et al. (2018)	Impulsivity
	Labouvie and McGee (1986)	Impulsivity
	Littlefield et al. (2009)	Impulsivity
	Littlefield, Sher, and Steinley (2010)	Impulsivity
	Littlefield and Sher (2012)	Impulsivity
	Littlefield et al. (2012)	Impulsivity
	Malmberg et al. (2013)	Impulsivity
	Mathijssen et al. (2021)	Impulsivity
	Quinn and Harden (2013)	Impulsivity
	Quinn et al. (2011)	Impulsivity
	Riley et al. (2018)	Impulsivity
	Robert et al. (2020)	Impulsivity
	White et al. (2011)	Impulsivity
	Wright and Jackson (2022)	Impulsivity
	Ashenhurst et al. (2015)	Sensation Seeking
	De Win et al. (2006)	Sensation Seeking
	Gmel et al. (2020)	Sensation Seeking

	Horvath et al. (2004)	Sensation Seeking
	Kaiser et al. (2016)	Sensation Seeking
	Kaiser et al. (2018)	Sensation Seeking
	Malmberg et al. (2013)	Sensation Seeking
	Mathijssen et al. (2021)	Sensation Seeking
	Quinn and Harden (2013)	Sensation Seeking
	Quinn et al. (2011)	Sensation Seeking
	Robert et al. (2020)	Sensation Seeking
	Wright and Jackson (2022)	Sensation Seeking
**Other**	Chassin et al. (2010)	Psychosocial Maturity
	De Moor et al. (2022)	Identity Status (Education and relational identity)
	Labouvie and McGee (1986)	Autonomy
	Quinn et al. (2011)	Autonomy
	Labouvie and McGee (1986)	Cognitive Structure

Table S3b

Personality trait clusters intervention studies

Cluster	Study included in review	Personality construct
**Extraversion and related traits**	Borman et al. (2006)	Extraversion
	Carter et al. (2001)	Extraversion
	Oei and Jackson (1980)	Extraversion
	Piedmont et al. (2001)	Extraversion
	Stieger et al. (2022)	Extraversion
	Blonigen and Macia (2021)	Positive Emotionality
**Neuroticism and related traits**	Borman et al. (2006)	Neuroticism
	Carter et al. (2001)	Neuroticism
	Oei and Jackson (1980)	Neuroticism
	Piedmont et al. (2001)	Neuroticism
	Blonigen and Macia (2021)	Negative Emotionality
	Kazemi et al. (2014)	Anxiety Sensitivity
	Borman et al. (2006)	Harm-Avoidance
	Kazemi et al. (2014)	Introversion/hopelessness
**Openness and related traits**	Borman et al. (2006)	Openness
	Carter et al. (2001)	Openness
	Piedmont et al. (2001)	Openness
	Borman et al. (2006)	Novelty Seeking
	Borman et al. (2006)	Self-Transcendence
	Stieger et al. (2022)	Originality
**Conscientiousness and related traits**	Borman et al. (2006)	Conscientiousness
	Carter et al. (2001)	Conscientiousness
	Piedmont et al. (2001)	Conscientiousness
	Stieger et al. (2022)	Conscientiousness
	Blonigen and Macia (2021)	Constraint
	Borman et al. (2006)	Persistence
	Borman et al. (2006)	Self-Directedness
**Agreeableness and related traits**	Borman et al. (2006)	Agreeableness
	Carter et al. (2001)	Agreeableness
	Piedmont et al. (2001)	Agreeableness
	Stieger et al. (2022)	Agreeableness
	Borman et al. (2006)	Cooperativeness
	Stieger et al. (2022)	Honesty
**Self-efficacy and related traits**	Bayır and Aylaz (2021)	Self-efficacy
	Can Gür and Okanli (2019)	Self-efficacy
	Chodkiewicz and Gruszczyńska (2019)	Self-efficacy
	Kayaoğlu and Şahin Altun (2022)	Self-efficacy
	Winkleby, Feighery, Altman, Kole, and Tencati (2001)	Self-efficacy
	Winkleby et al. (2004)	Self-efficacy
	Nurco et al. (1995)	Locus of control
**Impulsivity and related traits**	Aklin et al. (2009)	Impulsiveness
	Goncalves et al. (2014)	Impulsivity
	Kazemi et al. (2014)	Impulsivity
	Littlefield et al. (2015)	Impulsivity
	Aklin et al. (2009)	Risk Taking Propensity
	Kazemi et al. (2014)	Sensation Seeking
	Littlefield et al. (2015)	Sensation Seeking
**Other**	Borman et al. (2006)	Reward Dependence
	Oei and Jackson (1980)	Psychoticism
	Stieger et al. (2 022)	Resilience

Table S4

Directionality of change

Directionality of change in observational studies. Of the 38 observational studies, 14 aimed to explicitly test directional associations between SU and personality changes within one model (mostly using cross-lagged panel models). Except for De Moor, Sijtsema, Weller, and Klimstra (2022), who found no overall association between SU and changing identity status, 13 studies reported bidirectional associations between personality traits and SU (Chassin et al., 2010; Gmel et al., 2020; Horvath, Milich, Lynam, Leukefeld, & Clayton, 2004; Kaiser, Bonsu, Charnigo, Milich, & Lynam, 2016; Kaiser, Davis, Milich, Smith, & Charnigo, 2018; Littlefield et al., 2012; Malmberg et al., 2013; Quinn & Harden, 2013; Quinn, Stappenbeck, & Fromme, 2011; Robert et al., 2020; Roberts & Bogg, 2004; Samek et al., 2018; Stein et al., 1987). Of these, there was slightly more evidence for pathways from personality to later SU than from SU to later personality (change). To illustrate, Littlefield et al. (2012) found bidirectional associations between personality and alcohol use only when shorter time frames were investigated. Across longer time frames, personality predicted alcohol use but not vice versa. Samek et al. (2018) posited that directionality may depend on the developmental time frame: They found bidirectional associations from ages 24 to 29, whereas from ages 17 to 24, only the pathway from personality to later alcohol use disorder was significant. In summary, previous research suggests bidirectional associations between SU and personality change. Depending on the age range, personality trait, and substance assessed, there is somewhat more evidence that personality predicts later SU than vice versa.

Table S5

Heterogeneity/comparability of studies

Samples

The sample sizes of the studies varied considerably, which may partly explain the inconsistent results. For example, large sample sizes are needed to detect small effects, so weak associations may have been significant in larger samples but not in smaller ones.

About one fifth of the observational studies was based on nationally representative samples, while the majority was based on selective (e.g., convenience) samples. Almost all population-based studies referred to the general population, while only a few referred to specific high-risk groups (e.g., male juvenile offenders). The intervention studies mostly consisted of selective samples of individuals with SUD (e.g., patients at an inpatient treatment center). Findings from selected samples are prone to sample bias and might be less generalizable (e.g., to individuals with lower treatment motivation who do not seek treatment). Moreover, studies were conducted in different countries and cultures, but mostly in Europe, North America, and Australia. Additional studies are needed to focus on other regions (e.g., South America, Africa, and Asia) and to systematically examine cross-cultural differences.

Study designs

In the observational studies, the number of assessments (2 to 14), study duration (1.5 to 30 years), and time between waves (0.5 to 20 years) varied widely, which may explain the heterogenous results. The intervention studies were often characterized by short study periods and did not always include additional follow-up assessments after the immediate post-assessment. However, additional follow-ups would be necessary to demonstrate personality change: Personality traits are conceptualized as stable in the short term, so that trait changes can only be shown if they persist over a longer period of time (e.g., several months). In particular, studies with multiple follow-ups would be advantageous to examine how such changes unfold. Based on repeated monthly follow-ups, one could test for linear and non-linear changes and investigate, for example, whether the reduction in neuroticism slows down or accelerates over time.

Assessments

All studies relied on self-reported measures of SU and personality, which may be subject to response and recall bias and may not accurately reflect participants' actual behaviors or characteristics. For example, individuals with severe SU may be prone to underreporting their consumption. Therefore, additional studies using other assessment methods (e.g., behavioral observations and other-reports) would be highly useful. In addition, a large range of different measures have been used to assess the same personality traits or SU behaviors, limiting the comparability across studies. Several studies even used self-constructed measures or selected individual items from existing scales. Thus, the psychometric properties of these measures remain unknown, limiting the interpretability of the results. In many intervention studies, personality traits were assessed as secondary outcomes (rather than primary outcomes), and thus their analysis was limited.

References

Allen, M. S., Vella, S. A., & Laborde, S. (2015). Health-related behaviour and personality trait development in adulthood. *Journal of Research in Personality*, *59*, 104–110.

Ashenhurst, J. R., Harden, K. P., Corbin, W. R., & Fromme, K. (2015). Trajectories of binge drinking and personality change across emerging adulthood. *Psychology of Addictive Behaviors*, *29*(4), 978.

Bayır, B., & Aylaz, R. (2021). The effect of mindfulness-based education given to individuals with substance-use disorder according to self-efficacy theory on self-efficacy perception. *Applied Nursing Research : ANR*, *57*, 151354. https://doi.org/10.1016/j.apnr.2020.151354

Blonigen, D. M., Durbin, C. E., Hicks, B. M., Johnson, W., McGue, M., & Iacono, W. G. (2015). Alcohol use initiation is associated with changes in personality trait trajectories from early adolescence to young adulthood. *Alcoholism: Clinical and Experimental Research*, *39*(11), 2163–2170. https://doi.org/10.1111/acer.12878

Blonigen, D. M., & Macia, K. S. (2021). Personality change during substance use disorder treatment is associated with improvements in abstinence self-efficacy post-treatment among US military veterans. *Journal of Substance Abuse Treatment*, *120*, 108187.

Borman, P. D., Zilberman, M. L., Tavares, H., Surís, A. M., El-Guebaly, N., & Foster, B. (2006). Personality changes in women recovering from substance-related dependence. *Journal of Addictive Diseases*, *25*(4), 59–66.

Can Gür, G., & Okanli, A. (2019). The Effects of Cognitive-Behavioral Model-Based Intervention on Depression, Anxiety, and Self-Efficacy in Alcohol Use Disorder. *Clinical Nursing Research*, *28*(1), 52–78. https://doi.org/10.1177/1054773817722688

Carter, J. A., Herbst, J. H., Stoller, K. B., van King, L., Kidorf, M. S., Costa Jr, P. T., & Brooner, R. K. (2001). Short-term stability of NEO–PI–R personality trait scores in opioid-dependent outpatients. *Psychology of Addictive Behaviors*, *15*(3), 255.

Chassin, L., Dmitrieva, J., Modecki, K., Steinberg, L., Cauffman, E., Piquero, A. R., . . . Losoya, S. H. (2010). Does adolescent alcohol and marijuana use predict suppressed growth in psychosocial maturity among male juvenile offenders? *Psychology of Addictive Behaviors*, *24*(1), 48–60. https://doi.org/10.1037/a0017692

Chodkiewicz, J., & Gruszczyńska, E. (2019). Changes in well-being, self-efficacy, and coping strategies during residential treatment of alcohol-addicted patients. *Roczniki Psychologiczne*, *16*(1), 85–105.

De Moor, E. L. de, Sijtsema, J. J., Weller, J. A., & Klimstra, T. A. (2022). Longitudinal links between identity and substance use in adolescence. *Self and Identity*, *21*(1), 113–136. https://doi.org/10.1080/15298868.2020.1818615

Gmel, G., Marmet, S., Studer, J., & Wicki, M. (2020). Are changes in personality traits and alcohol use associated? A cohort study among young Swiss men. *Frontiers in Psychiatry*, *11*, 591003.

Hakulinen, C., & Jokela, M. (2019). Alcohol use and personality trait change: Pooled analysis of six cohort studies. *Psychological Medicine*, *49*(2), 224–231. https://doi.org/10.1017/s0033291718000636

Hemphälä, M., Kosson, D., Westerman, J., & Hodgins, S. (2015). Stability and predictors of psychopathic traits from mid-adolescence through early adulthood. *Scandinavian Journal of Psychology*, *56*(6), 649–658. https://doi.org/10.1111/sjop.12257

Hicks, B. M., Durbin, C. E., Blonigen, D. M., Iacono, W. G., & McGue, M. (2012). Relationship between personality change and the onset and course of alcohol dependence in young adulthood. *Addiction*, *107*(3), 540–548. https://doi.org/10.1111/j.1360-0443.2011.03617.x

Horvath, L. S., Milich, R., Lynam, D., Leukefeld, C., & Clayton, R. (2004). Sensation seeking and substance use: a cross-lagged panel design. *Individual Differences Research*, *2*(3).

Jokela, M., Airaksinen, J., Kivimäki, M., & Hakulinen, C. (2018). Is Within–Individual Variation in Personality Traits Associated with Changes in Health Behaviours? Analysis of Seven Longitudinal Cohort Studies. *European Journal of Personality*, *32*(6), 642–652. https://doi.org/10.1002/per.2173

Kaiser, A. J., Bonsu, J. A., Charnigo, R. J., Milich, R., & Lynam, D. R. (2016). Impulsive personality and alcohol use: Bidirectional relations over one year. *Journal of Studies on Alcohol and Drugs*, *77*(3), 473–482. https://doi.org/10.15288/jsad.2016.77.473

Kaiser, A. J., Davis, H. A., Milich, R., Smith, G. T., & Charnigo, R. (2018). Bidirectional relations of impulsive personality and alcohol use across three waves of data collection. *Substance Use & Misuse*, *53*(14), 2386–2393. https://doi.org/10.1080/10826084.2018.1480036

Kayaoğlu, K., & Şahin Altun, Ö. (2022). The effect of combined cognitive-behavioral psychoeducation and music intervention on stress, self-efficacy, and relapse rates in patients with alcohol and substance use disorders: A randomized controlled trial. *Perspectives in Psychiatric Care*, *58*(3), 968–977. https://doi.org/10.1111/ppc.12884

Kazemi, D. M., Levine, M. J., Dmochowski, J., Angbing, I., & Shou, Q. (2014). Personality risk factors and readiness to change in mandated and voluntary college students enrolled in an alcohol intervention program. *Substance Use & Misuse*, *49*(1-2), 154–165. https://doi.org/10.3109/10826084.2013.824472

Klimstra, T. A., Luyckx, K., Hale, W. W., & Goossens, L. (2014). Personality and externalizing behavior in the transition to young adulthood: The additive value of personality facets. *Social Psychiatry and Psychiatric Epidemiology*, *49*(8), 1319–1333. https://doi.org/10.1007/s00127-014-0827-y

Kroencke, L., Kuper, N., Bleidorn, W., & Denissen, J. (2021). How does substance use affect personality development? Disentangling between- and within-person effects. *Social Psychological and Personality Science*, *12*(4), 517–527. https://doi.org/10.1177/1948550620921702

Labouvie, E. W., & McGee, C. R. (1986). Relation of personality to alcohol and drug use in adolescence. *Journal of Consulting and Clinical Psychology*, *54*(3), 289–293. https://doi.org/10.1037/0022-006x.54.3.289

Littlefield, A. K., & Sher, K. J. (2012). Smoking desistance and personality change in emerging and young adulthood. *Nicotine & Tobacco Research*, *14*(3), 338–342. https://doi.org/10.1093/ntr/ntr219

Littlefield, A. K., Sher, K. J., & Steinley, D. (2010). Developmental trajectories of impulsivity and their association with alcohol use and related outcomes during emerging and young adulthood I. *Alcoholism, Clinical and Experimental Research*, *34*(8), 1409–1416. https://doi.org/10.1111/j.1530-0277.2010.01224.x

Littlefield, A. K., Sher, K. J., & Wood, P. K. (2009). Is "maturing out" of problematic alcohol involvement related to personality change? *Journal of Abnormal Psychology*, *118*(2), 360.

Littlefield, A. K., Sher, K. J., & Wood, P. K. (2010). A personality-based description of maturing out of alcohol problems: Extension with a five-factor model and robustness to modeling challenges. *Addictive Behaviors*, *35*(11), 948–954.

Littlefield, A. K., Vergés, A., Wood, P. K., & Sher, K. J. (2012). Transactional models between personality and alcohol involvement: A further examination. *Journal of Abnormal Psychology*, *121*(3), 778.

Luchetti, M., Terracciano, A., Stephan, Y., & Sutin, A. R. (2018). Alcohol use and personality change in middle and older adulthood: Findings from the Health and Retirement Study. *Journal of Personality*, *86*(6), 1003–1016. https://doi.org/10.1111/jopy.12371

Malmberg, M., Kleinjan, M., Overbeek, G., Vermulst, A. A., Lammers, J., & Engels, R. C. (2013). Are there reciprocal relationships between substance use risk personality profiles and alcohol or tobacco use in early adolescence? *Addictive Behaviors*, *38*(12), 2851–2859. https://doi.org/10.1016/j.addbeh.2013.08.003

Mathijssen, J. J., Rozema, A. D., Hiemstra, M., Jansen, M. W., & van Oers, J. A. (2021). Stability of and change in substance use risk personality: Gender differences and smoking cigarettes among early adolescents. *Addictive Behaviors Reports*, *14*, 100360.

Nurco, D. N., Primm, B. J., Lerner, M., Stephenson, P., Brown, L. S., & Ajuluchukwu, D. C. (1995). Changes in locus-of-control attitudes about drug misuse in a self-help group in a methadone

maintenance clinic. *International Journal of the Addictions*, *30*(6), 765–778. https://doi.org/10.3109/10826089509048758

Östlund, A., Hensing, G., Sundh, V., & Spak, F. (2007). Changes in some personality traits after recovery from alcohol dependence/abuse, anxiety and depression—Results of a 5-year follow-up in a general population sample of women. *Nordic Journal of Psychiatry*, *61*(4), 279–287. https://doi.org/10.1080/08039480701415111

Piedmont, R. L. (2001). Cracking the plaster cast: Big Five personality change during intensive outpatient counseling. *Journal of Research in Personality*, *35*(4), 500–520.

Quinn, P. D., & Harden, K. P. (2013). Differential changes in impulsivity and sensation seeking and the escalation of substance use from adolescence to early adulthood. *Development and Psychopathology*, *25*(1), 223–239. https://doi.org/10.1017/s0954579412000284

Quinn, P. D., Stappenbeck, C. A., & Fromme, K. (2011). Collegiate heavy drinking prospectively predicts change in sensation seeking and impulsivity. *Journal of Abnormal Psychology*, *120*(3), 543–556. https://doi.org/10.1037/a0023159

Robert, G. H., Luo, Q., Yu, T., Chu, C., Ing, A., Jia, T., . . . Schumann, G. (2020). Association of gray matter and personality development with increased drunkenness frequency during adolescence. *JAMA Psychiatry*, *77*(4), 409. https://doi.org/10.1001/jamapsychiatry.2019.4063

Roberts, B. W., & Bogg, T. (2004). A longitudinal study of the relationships between conscientiousness and the social-environmental factors and substance-use behaviors that influence health. *Journal of Personality*, *72*(2), 325–354. https://doi.org/10.1111/j.0022-3506.2004.00264.x

Samek, D. R., Hicks, B. M., Durbin, E., Hinnant, J. B., Iacono, W. G., & McGue, M. (2018). Codevelopment between key personality traits and alcohol use disorder from adolescence through young adulthood. *Journal of Personality*, *86*(2), 261–282. https://doi.org/10.1111/jopy.12311

Stein, J. A., Newcomb, M. D., & Bentler, P. M. (1987). Personality and drug use: reciprocal effects across four years. *Personality and Individual Differences*, *8*(3), 419–430.

Stephan, Y., Sutin, A. R., Luchetti, M., Caille, P., & Terracciano, A. (2019). Cigarette smoking and personality change across adulthood: Findings from five longitudinal samples. *Journal of Research in Personality*, *81*, 187–194. https://doi.org/10.1016/j.jrp.2019.06.006

Stieger, M., Allemand, M., Roberts, B. W., & Davis, J. P. (2022). Mindful of personality trait change: Are treatment effects on personality trait change ephemeral and attributable to changes in states? *Journal of Personality*, *90*(3), 375–392.

Turiano, N. A., Whiteman, S. D., Hampson, S. E., Roberts, B. W., & Mroczek, D. K. (2012). Personality and substance use in midlife: Conscientiousness as a moderator and the effects of trait change. *Journal of Research in Personality*, *46*(3), 295–305. https://doi.org/10.1016/j.jrp.2012.02.009

Welch, D., & Poulton, R. (2009). Personality influences on change in smoking behavior. *Health Psychology: Official Journal of the Division of Health Psychology, American Psychological Association*, *28*(3), 292–299. https://doi.org/10.1037/a0013471

White, H. R., Marmorstein, N. R., Crews, F. T., Bates, M. E., Mun, E.-Y., & Loeber, R. (2011). Associations between heavy drinking and changes in impulsive behavior among adolescent boys. *Alcoholism, Clinical and Experimental Research*, *35*(2), 295–303. https://doi.org/10.1111/j.1530-0277.2010.01345.x

Winkleby, M. A., Feighery, E. C., Altman, D. A., Kole, S. [S.], & Tencati, E. (2001). Engaging ethnically diverse teens in a substance use prevention advocacy program. *American Journal of Health Promotion : AJHP*, *15*(6), 433-6, ii. https://doi.org/10.4278/0890-1171-15.6.433

Winkleby, M. A., Feighery, E., Dunn, M., Kole, S. [Sara], Ahn, D., & Killen, J. D. (2004). Effects of an advocacy intervention to reduce smoking among teenagers. *Archives of Pediatrics & Adolescent Medicine*, *158*(3), 269–275. https://doi.org/10.1001/archpedi.158.3.269
